# A vitamin B_3_–driven root bacterial metabolite primes systemic immunity in *Arabidopsis*

**DOI:** 10.1126/sciadv.aef8959

**Published:** 2026-08-26

**Authors:** Xuemei Wang, Peng Jiang, Xindan Xu, Jingfang Zhang, Guiyang Xia, Wenhan Qian, Qingwen Chen, Jin-Wei Wei, Zhuo Liu, Lingyun Liu, Fengxia Zhang, Lei Li, Sheng Lin, Yang Bai, Guodong Wang

**Affiliations:** ^1^ State Key Laboratory of Seed Innovation, Institute of Genetics and Developmental Biology, Chinese Academy of Sciences, Beijing 100101, China.; ^2^ State Key Laboratory of Gene Function and Modulation Research, Peking-Tsinghua Center for Life Sciences, Peking-Tsinghua-NIBS Graduate Program, College of Life Sciences, Peking University, Beijing 100871, China.; ^3^ School of Pharmacy, Shanxi Medical University, Taiyuan 030001, China.; ^4^ Key Laboratory of Chinese Internal Medicine of Ministry of Education and Beijing, Dong-zhi-men Hospital, Beijing University of Chinese Medicine, Beijing 100700, China.; ^5^ School of Chinese Materia Medica, Beijing University of Chinese Medicine, Beijing 100029, China.; ^6^ College of Advanced Agricultural Sciences, Chinese Academy of Sciences, Beijing 100049, China.

## Abstract

The microbiota is being increasingly recognized for its ability to regulate host physiology through the production of small bioactive molecules. However, how host-derived nutrients are metabolically transformed by root-associated microbes to influence plant immunity remains poorly understood. Here, we show that vitamin B_3_ (VB_3_; niacin) secreted by plant roots shapes the assembly of a functionally specialized root microbiota, which, in turn, metabolizes VB_3_ into an immune-active signal that enhances plant disease resistance. VB_3_ secretion selectively increases the abundance of root-associated bacteria harboring a conserved *nic* biosynthetic gene cluster (BGC), which enables the conversion of VB_3_ into 6-hydroxynicotinate (6-OHNA), a previously uncharacterized microbial metabolite involved in plant-microbe interactions. Microbially produced 6-OHNA is transported from roots to shoots, where it primes systemic immune responses in a salicylic acid–dependent manner. Disruption of the microbial *nic* BGC abolishes immune priming, whereas increased VB_3_ exudation from plant roots enhances disease resistance. Together, these findings reveal a metabolically mediated dialog between plant hosts and their microbiota that links host nutrient secretion to microbial functional specialization and the activation of systemic plant immunity.

## INTRODUCTION

Vitamin B_3_ (VB_3_), in the form of nicotinate (NA) and nicotinamide (NAM), serves as a universal precursor of nicotinamide adenine dinucleotide (NAD), a ubiquitous coenzyme that fuels metabolic pathways and orchestrates a broad spectrum of biological processes across all forms of life (*1*–*5*). In most organisms, including microbes, animals, and plants, NAD biosynthesis proceeds primarily via two conserved pathways: the de novo pathway, which originates from aspartate (Asp) or tryptophan, and the salvage pathway, which recycles VB_3_ intermediates (*6*, *7*). Both routes are indispensable for normal plant growth and development (*8*–*10*).

Beyond NAD biosynthesis, VB_3_ modifications act as an additional regulatory layer for maintaining NAD homeostasis under fluctuating physiological and environmental conditions. Although VB_3_ modification has rarely been documented outside plants, aside from the formation of 1-methylnicotinamide, a metabolite implicated in tumor progression in humans (*11*, *12*), plants have evolved a suite of strategies to chemically modify VB_3_, particularly NA, to prevent its overaccumulation. For example, methyl NA is transported from roots to shoots to support NAD synthesis (*13*). In contrast, NA *O*-glucoside (NA*O*G) accumulates intracellularly, whereas *N*-glucosides (NA*N*G) are secreted, thereby excluding NA from the NAD cycle (*13*, *14*). In addition, trigonelline (Tg; *N*-methyl NA) serves as a detoxification product of excess NA and contributes to NAD homeostasis (*15*).

In contrast to plants and mammals, microbes exhibit markedly diverse in NAD biosynthetic strategies. Some species cannot even synthesize NAD de novo and instead rely on scavenging VB_3_ from their environment, often from host organisms (*16*). Beyond serving as an NAD precursor, certain microbes can degrade VB_3_ and use it as a direct carbon and nitrogen source (*17*–*21*). Recent work has revealed trans-kingdom NAD-boosting cooperation in which gut bacteria supply NA that fuels host NAD synthesis via the salvage pathway (*22*, *23*). Even bacteriophages have evolved alternative NAD-generating mechanisms that convert adenosine 5′-diphosphate–ribose and NAM into NAD during infection (*24*).

The root niche, a dynamic interface between plant roots and soil microbes, forms a complex holobiont that profoundly influences plant growth, health, and stress adaptation (*25*, *26*). Root-secreted metabolites, including both primary and specialized compounds, play pivotal roles in shaping microbial community assembly (*27*–*32*). Conversely, the root microbiota enhances host nutrition through processes such as nitrogen fixation and phosphate solubilization and activates immune defenses (*33*–*35*). The advent of in situ metabolome-microbiome profiling, together with synthetic community approaches, now enables mechanistic dissection of these bidirectional interactions (*36*). However, the ecological roles of plant-derived VB_3_ in the root microenvironment, for both the host plant and root-associated microbiota, remain largely unexplored.

Although VB_3_-related metabolites have been detected in root exudates, whether they influence root microbiome assembly and whether root-associated microbes further transform these compounds into bioactive signals that affect host physiology remain unknown. Here, we sought to determine how plant-derived VB_3_ shapes root microbiome assembly, whether root-associated bacteria metabolize VB_3_ into previously unrecognized compounds, and whether such microbially derived metabolites affect host responses. To address these questions, we compared the root microbiomes and VB_3_-related exudate profiles of *Arabidopsis thaliana* mutants defective in NA biosynthesis or modification (*nic1-1*, *ugt74f2*, and *ugt74f2 ugt76c4*) with those of wild-type Columbia-0 (Col-0). We found that host-derived VB_3_ profoundly shaped the microbial community composition. Unexpectedly, we identified specific root-associated bacteria capable of converting NA into 6-OHNA, a previously uncharacterized microbial metabolite. Functional analyses further revealed that 6-OHNA enhances resistance to pathogen infection by priming plant immunity, revealing a previously unrecognized mechanism through which the microbial metabolism of host-derived nutrients feeds back to regulate host defense.

## RESULTS

### Distinct VB_3_ exudation patterns reshape the root microbiota in *Arabidopsis* mutants

**Fig. 1.**
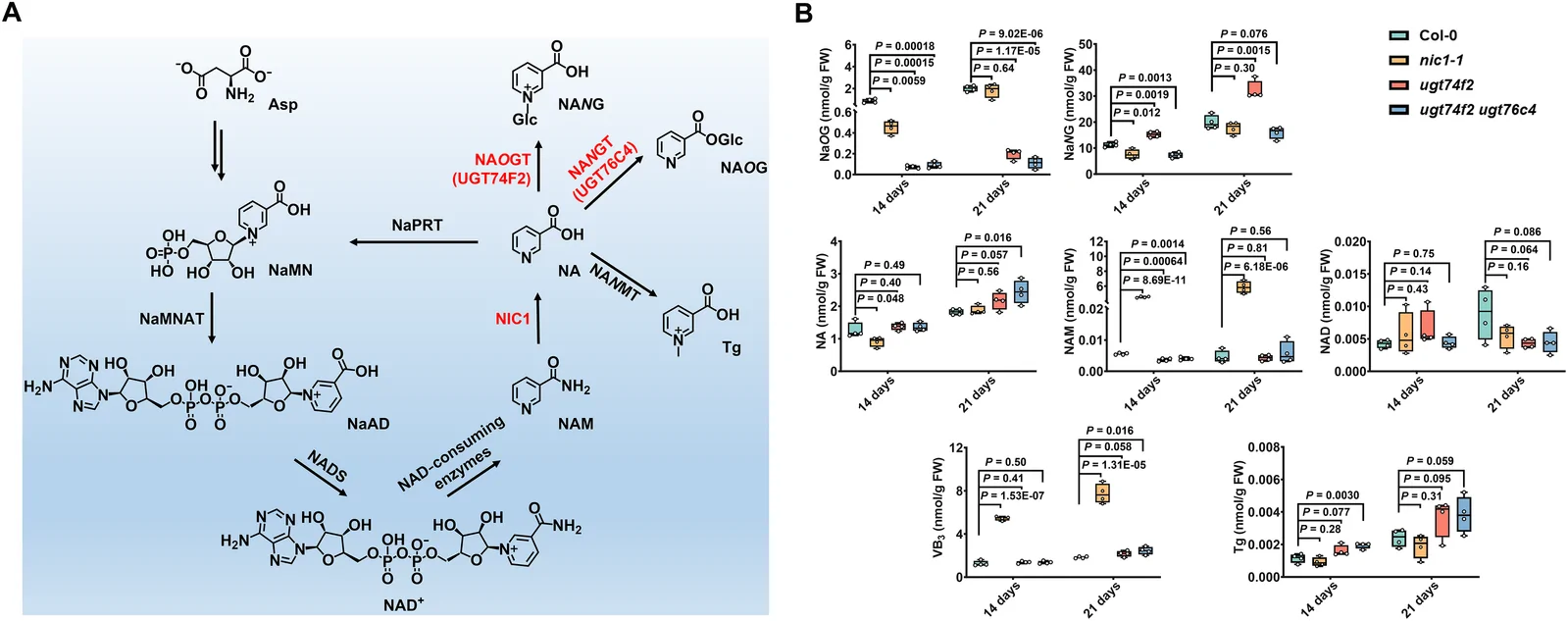
Profiling of VB_3_-related metabolites in root exudates of *Arabidopsis* NA homeostasis mutants. (**A**) Schematic representation of NA metabolic and homeostasis pathway in *Arabidopsis*, with key enzymes catalyzing each reaction step annotated. NaMN, nicotinate mononucleotide; NaAD, nicotinate adenine dinucleotide; NIC1, nicotinamidase 1; NaPRT, nicotinate phosphoribosyltransferase; NaMNAT, nicotinate mononucleotide adenylyltransferase; NADS, NAD synthase; NA*O*GT (UGT74F2), nicotinate *O*-glycosyltransferase; NA*N*GT (UGT76C4), nicotinate *N*-glycosyltransferase; NA*N*MT, nicotinate *N*-methyltransferase. (**B**) Quantification of VB_3_-related metabolites in root exudates of Col-0 and NA homeostasis–related mutants. VB_3_, sum of NA and NAM. FW, fresh weight. Data are presented as box plots with individual data points (*n* = 4). Statistical significance was assessed using unpaired, two-tailed Student’s *t* tests; *P* values are shown. Similar results were obtained in three independent experiments.

To assess how VB_3_ (NA and NAM) metabolism influences rhizosphere plant-microbe interactions, we analyzed *Arabidopsis* mutants with impaired NA biosynthesis (*nic1-1*) or NA glycosylation (*ugt74f2* and *ugt74f2 ugt76c4*). Targeted metabolite profiling revealed pronounced genotype-dependent alterations in the secretion of NAD-pathway metabolites, including VB_3_. The *nic1-1* mutant, which is defective in nicotinamidase activity, secreted markedly reduced amounts of NA but accumulated high levels of NAM, accompanied by decreased levels of glycosylated derivatives. Consistent with these changes, *nic1-1* secreted a total of 7.73 ± 0.87 nmol g^−1^ of VB_3_ metabolites compared with Col-0 (1.83 ± 0.08 nmol g^−1^), representing a >4.2-fold increase. In contrast, *ugt74f2* exhibited a selective redistribution among NA glycosylation products without a substantial change in total VB_3_ output, whereas the *ugt74f2 ugt76c4* double mutant exhibited a broad loss of glycosylated forms together with a pronounced accumulation of free NA. Accordingly, compared with Col-0, *ugt74f2 ugt76c4* also significantly increased VB_3_ secretion (2.45 ± 0.36 nmol g^−1^), corresponding to an approximately 1.3-fold increase. Together, these results indicate that disruption of either NA biosynthesis or dual glycosylation rebalances metabolic flux toward increased VB_3_ release into the rhizosphere (Fig. 1, A and B).

**Fig. 2.**
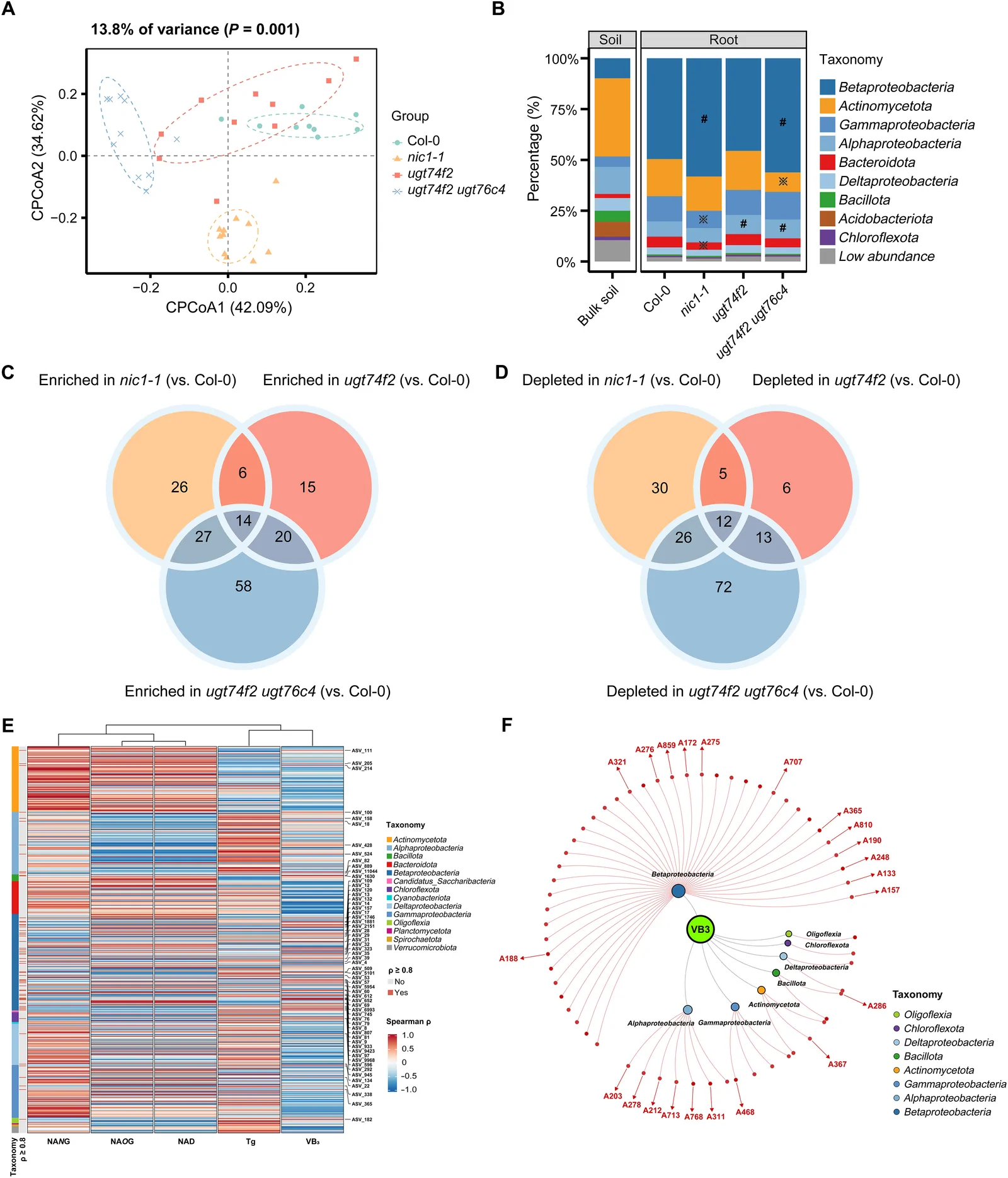
Root microbiota alterations associated with *Arabidopsis* NA homeostasis mutants. (**A**) Constrained principal coordinate analysis (CPCoA) based on Bray-Curtis dissimilarity showing distinct root-associated bacterial communities across genotypes. Each point represents a biological replicate, with colors indicating genotypes. Ellipses denote 68% confidence intervals. Sample sizes: Col-0 (*n* = 9), *nic1-1* (*n* = 11), *ugt74f2* (*n* = 10), and *ugt74f2 ugt76c4* (*n* = 10). (**B**) Phylum-level composition of root-associated microbiota across genotypes. *Pseudomonadota* is further resolved at the class level. Asterisks (*) indicate a significant decrease, and pound signs (#) indicate a significant increase (*P* < 0.05) relative to Col-0. Bulk soil; *n* = 6. (**C** and **D**) Venn diagrams showing ASVs enriched (C) or depleted (D) in the root microbiota of *nic1-1*, *ugt74f2*, and *ugt74f2 ugt76c4* relative to Col-0. (**E**) Heatmap showing Spearman correlations between NA-related metabolites and differentially abundant root bacterial ASVs. Rows represent ASVs, and columns represent metabolites (VB_3_, NA*O*G, NA*N*G, NAD, and Tg). ASVs showing strong positive correlations with VB_3_ (ρ ≥ 0.8) are highlighted. Taxonomic classifications are shown on the left. The color gradient indicates the strength and direction of the Spearman correlation coefficient (red = positive correlation, blue = negative correlation). (**F**) Co-occurrence network of VB_3_ and positively correlated root bacterial ASVs (ρ ≥ 0.8). Node colors indicate taxonomy at the phylum level, with *Pseudomonadota* resolved at the class level. Node size corresponds to the number of ASVs within each respective taxonomic group. ASVs matching cultured isolates [>97% 16*S* ribosomal RNA (rRNA) identity] are labeled.

We next asked whether these genotype-dependent differences in VB_3_ secretion influence root-associated microbial communities as previously described (*30*, *31*, *37*). Despite exhibiting no obvious differences in growth or biomass relative to those of Col-0 (fig. S1), the root microbiome of the VB_3_-related mutants underwent genotype-dependent restructuring. Multivariate analyses revealed significant separation between Col-0 and the VB_3_ mutants, with the plant genotype explaining 13.8% of the total variance (*P* = 0.001; permutational multivariate analysis of variance; Fig. 2A). Pairwise comparisons of community dissimilarity further demonstrated distinct genotype-dependent shifts in β-diversity. Notably, *nic1-1* and *ugt74f2 ugt76c4*, the two genotypes that exhibited elevated VB_3_ secretion, showed the most pronounced compositional divergence from Col-0, whereas *ugt74f2* largely retained a community structure similar to that of Col-0 (fig. S2). Consistent with previous observations, microbial α-diversity was reduced in roots compared with that in bulk soil, with *nic1-1* showing a further significant decrease among the mutants (fig. S3).

Taxonomic profiling further revealed distinct phylum-level differences between VB_3_ homeostasis mutants and Col-0. Given that *Pseudomonadota* accounted for more than 50% of the total community, the taxa within this phylum were further evaluated at the class level. The *nic1-1* mutant showed an increase in *Betaproteobacteria* abundance, accompanied by reductions in *Gammaproteobacteria* and *Bacteroidota* abundance (Fig. 2B). In contrast, both *ugt74f2* and *ugt74f2 ugt76c4* were markedly enriched in *Alphaproteobacteria*, while *ugt74f2 ugt76c4* uniquely exhibited a significant increase in *Betaproteobacteria* abundance together with a pronounced decrease in *Actinomycetota* abundance (Fig. 2B). Amplicon sequence variant (ASV)–level analyses reinforced these patterns by revealing both shared and genotype-specific recruitment signatures (figs. S4 and S5 and table S1, A and B). Notably, 14 ASVs were consistently enriched across all the mutants, whereas *nic1-1* and *ugt74f2 ugt76c4* each displayed genotype-specific enrichment of 41 ASVs, accounting for 56.2 and 34.5% of their respective differentially enriched taxa. These results highlight both convergent and divergent microbiome responses to disruption of VB_3_ metabolism (Fig. 2, C and D).

To directly link metabolite secretion with microbial recruitment, we constructed a co-occurrence network between the abundances of NA-derived metabolites and ASVs. This analysis revealed a distinct cluster of ASVs that was positively correlated with VB_3_ levels and largely independent of other VB_3_-related metabolites (Fig. 2, E and F, and table S1C). Notably, 25 of the 41 ASVs (61%) commonly enriched in *nic1-1* and *ugt74f2 ugt76c4* showed strong positive correlations with VB_3_ levels, indicating that elevated NA and NAM secretion are major drivers of shared microbial enrichment. Collectively, these data demonstrate that perturbation of NA metabolism reshapes root exudate composition and selectively results in the recruitment of distinct microbial lineages, with increased VB_3_ availability serving as a central ecological cue governing rhizosphere community assembly.

### Biosynthesis and secretion of 6-OHNA by VB_3_-associated bacteria

**Fig. 3.**
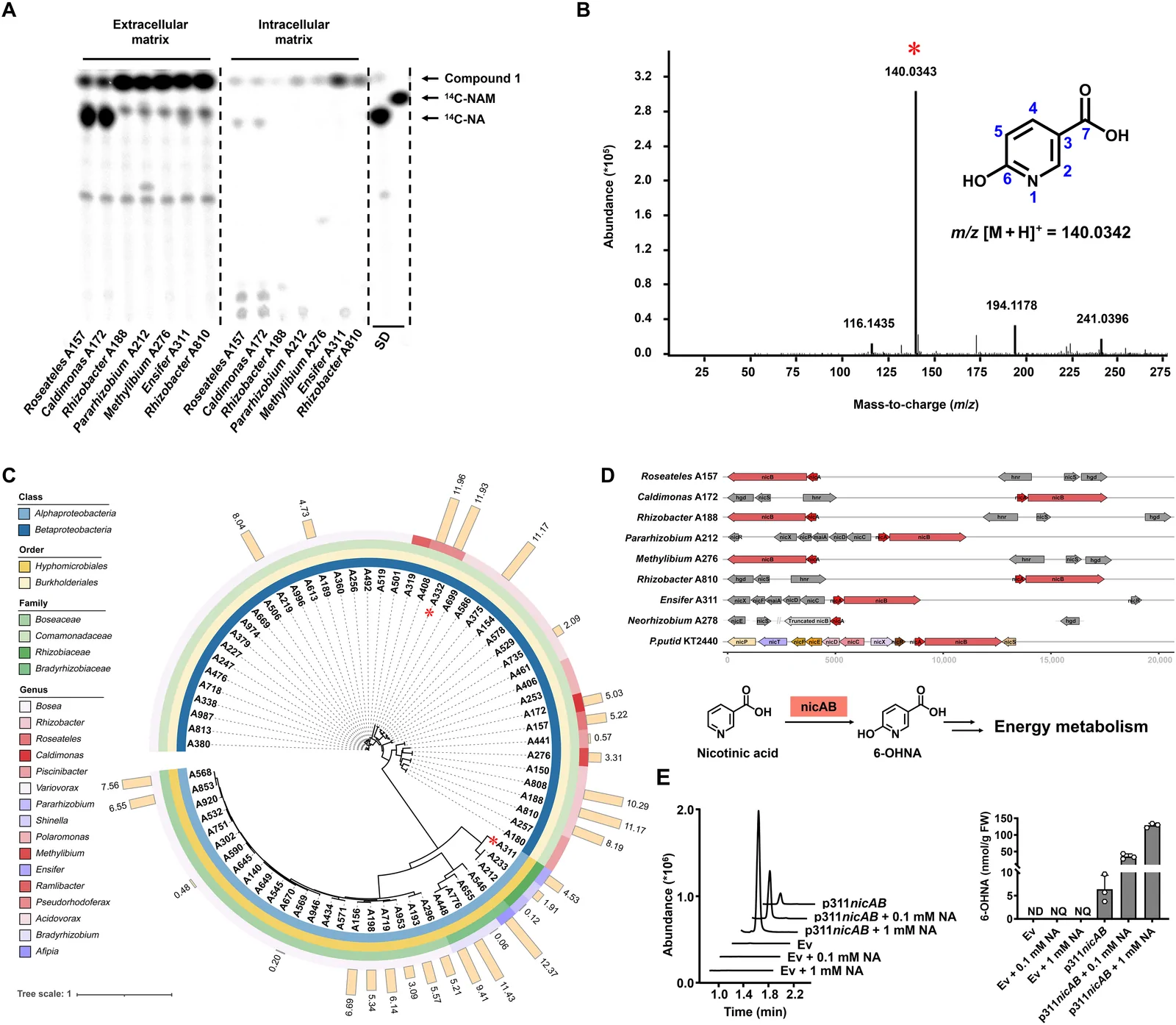
Identification, distribution, and biosynthesis of microbially derived 6-OHNA. (**A**) Thin-layer chromatography (TLC) analysis of VB_3_-associated bacterial strains incubated with [^14^C]-NA, showing conversion of NA into an uncharacterized metabolite (Compound 1). Migration positions of [^14^C]-NA and [^14^C]-NAM standards are indicated. (**B**) High-resolution mass spectrum and structural identification of Compound 1 as 6-hydroxynicotinate (6-OHNA). The major ion peak at mass/charge ratio (*m/z*) of [M + H]^+^ = 140.0343 is marked with an asterisk. (**C**) Taxonomic distribution and functional validation of *nicAB*-encoding strains in the *Arabidopsis* root microbiota. Thirty representative strains were assayed for 6-OHNA production following NA supplementation. Bars indicate extracellular 6-OHNA accumulation quantified by liquid chromatography–triple quadrupole–mass spectrometry (LC-QQQ-MS). Data are means ± SD (*n* = 3). *Ensifer* A311 and *Pseudorhodoferax* A332 are highlighted for subsequent analyses. (**D**) Organization of the *nic* BGC and the associated aerobic NA degradation pathway. Comparative BGC architectures of eight VB_3_-associated isolates and the reference strain *Pseudomonas putida* KT2440. *nicA* and *nicB* are highlighted in red; a truncated *nicB* in *Neorhizobium* A278 is shown in gray (top). Schematic of the bacterial aerobic NA degradation pathway, with *nicAB* highlighted as the putative NA 6-hydroxylase (bottom). (**E**) Transient expression of *nicAB* in *Nicotiana benthamiana* promotes 6-OHNA accumulation. Representative chromatograms (left) and metabolite quantification (right) are shown. Data are means ± SD (*n* = 3). Ev, empty vector; ND, not detected; NQ, not quantified.

Having identified bacterial taxa whose abundance is positively associated with rhizosphere VB_3_ abundance, we then investigated whether these bacteria actively metabolize plant-derived VB_3_ compounds. When incubated with [^14^C]-labeled NA or NAM, a subset of seven strains consistently converted VB_3_ into previously unannotated metabolites, most of which accumulated in the extracellular fraction (Fig. 3A and fig. S6). These strains spanned multiple genera, including *Roseateles*, *Caldimonas*, *Rhizobacter*, *Pararhizobium*, *Methylibium*, and *Ensifer*, indicating that this metabolic capability is phylogenetically widespread.

To identify these metabolites, we performed high-resolution liquid chromatography–quadrupole time-of-flight mass spectrometry (LC-qTOF/MS) on culture secretions from two representative strains, *Methylibium* A276 and *Ensifer* A311. Both strains produced a unique ion at mass/charge ratio (*m/z*) of 140.0343 ([M + H]^+^) in positive ionization mode, which was detected exclusively in NA-fed cultures (Fig. 3B and fig. S7, A and B). The 16-Da mass increase relative to that of NA (123.0315) suggests a hydroxylation reaction. Nuclear magnetic resonance (NMR) analysis of purified extracts from *Ensifer* A311 confirmed the presence of hydroxylation at the C6 position, as evidenced by a downfield shift of the corresponding ^13^C signal (*38*); thus, the metabolite was identified as 6-hydroxynicotinate (6-OHNA) (fig. S7, C and D, and table S2A). Notably, 6-OHNA accumulated predominantly in culture supernatants rather than within bacterial cells, suggesting that it is actively released into the extracellular environment and may therefore function as a microbially derived rhizosphere metabolite.

### The *nic* biosynthetic gene cluster underlies 6-OHNA production in VB_3_-associated root bacteria

To elucidate the molecular basis of VB_3_-mediated plant-microbe interactions, we used a well-established pipeline (*39*) to sequence and analyze the genomes of 441 bacterial isolates cultured from *Arabidopsis* roots. Genome mining revealed extensive biosynthetic potential, including diverse biosynthetic gene clusters (BGCs) encoding nonribosomal peptide synthetases (NRPSs), polyketide synthases (PKSs), ribosomally synthesized and posttranslationally modified peptides (RiPPs), terpenes, and other secondary metabolites (table S2B). Notably, the *nic* BGC, which mediates NA catabolism for bacterial energy metabolism (*19*, *20*), was detected in 16.6% (^73^/_441_) of the isolates (Fig. 3C and table S2C).

Functional assays of 30 *nic*-harboring strains randomly selected from 16 genera (Fig. 3C) demonstrated their ability to convert NA into 6-OHNA. However, the rate of 6-OHNA secretion varied markedly among the strains (Fig. 3C). The *nic* BGC was significantly enriched among VB_3_-associated bacteria, with 7 of 22 (31.8%) VB_3_-responsive isolates carrying *nic* (Fig. 3D and tables S1D and S2C). Time-resolved quantification of intra- and extracellular 6-OHNA in two representative *nic*-containing strains, *Ensifer* A311 and *Pseudorhodoferax* A332, revealed that more than 80% of the metabolite was secreted into the extracellular compartment at early time points (fig. S8). The initial step of this pathway is catalyzed by NicA/NicB (NicAB), a two-subunit NA hydroxylase originally characterized in *Pseudomonas putida* KT2440 and *Eubacterium barkeri*, which converts NA into 6-OHNA (Fig. 3D) (*19*, *20*).

Consistent with this model, seven of the eight representative *nic*-harboring isolates efficiently used NA or NAM as the sole carbon and nitrogen sources, whereas *Neorhizobium* A278 failed to do so, in line with its defective *nic* locus (fig. S9, A and B). Transient expression of *nicAB* in *Nicotiana benthamiana* leaves resulted in NA-dependent accumulation of 6-OHNA, establishing NicAB as the enzymatic driver of NA 6-hydroxylation (Fig. 3E). In planta colonization assays revealed preferential enrichment of strains carrying intact *nic* BGCs in VB_3_-accumulating mutants (*nic1-1* and *ugt74f2 ugt76c4*) relative to Col-0, with *Roseateles* A157 and *Rhizobacter* A810 consistently enriched across genotypes (fig. S9C).

### Microbially derived 6-OHNA primes salicylic acid–dependent immunity in *Arabidopsis*

**Fig. 4.**
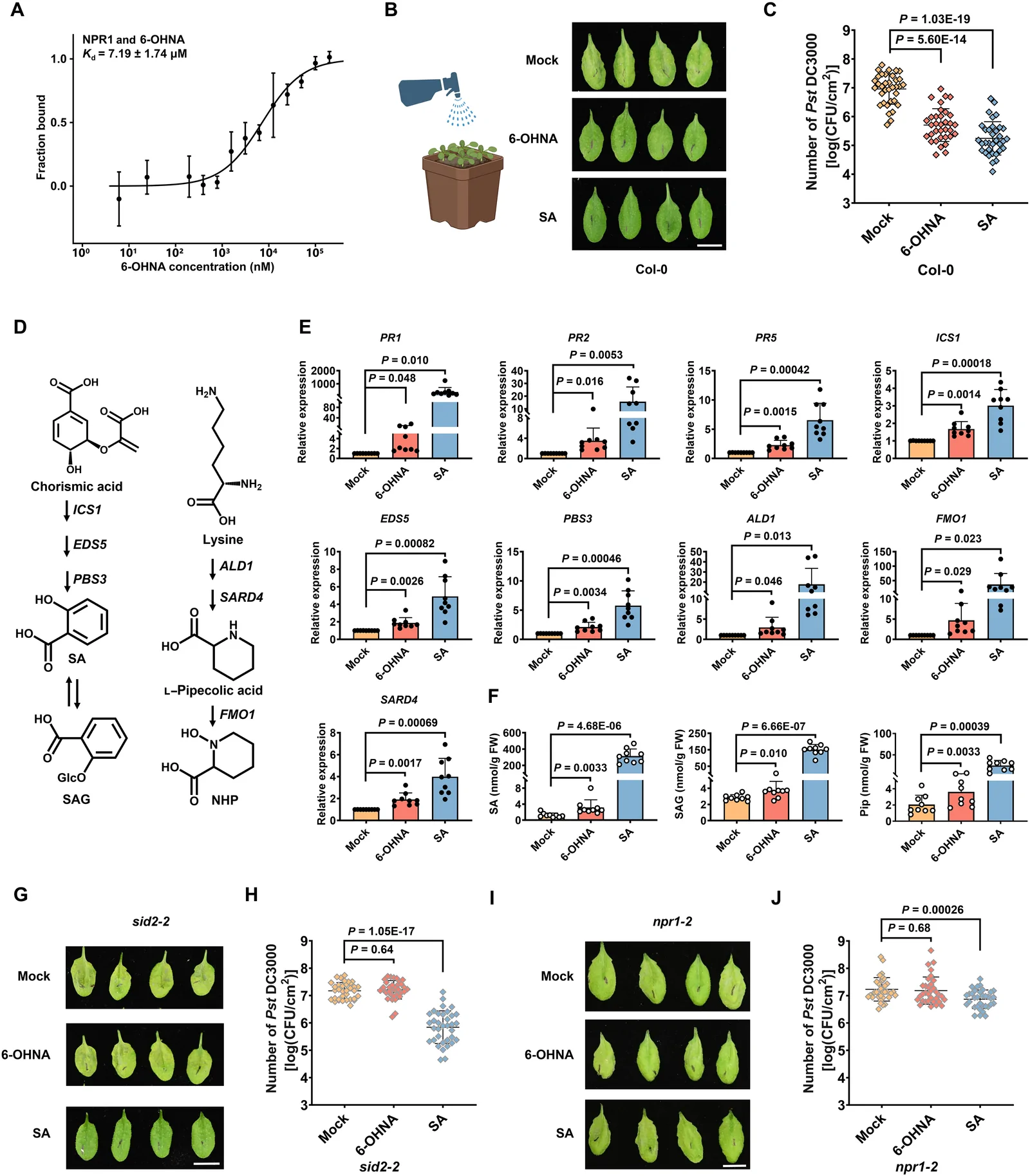
6-OHNA–triggered immune priming relies on intact SA biosynthesis and NPR1-mediated SA signaling in *Arabidopsis*. (**A**) Binding of 6-OHNA to the SA receptor NPR1 measured by microscale thermophoresis (MST), with *K*_d_ = 7.19 ± 1.74 μM, presented as mean ± SD (*n* = 3). (**B**) Foliar application of 6-OHNA enhances resistance to *Pst* DC3000. Col-0 plants were pretreated with 1 mM 6-OHNA, 1 mM SA (positive control), or dimethyl sulfoxide (DMSO; mock) and photographed at 3 days postinoculation (dpi). Representative images from three independent experiments are shown. Scale bar, 1 cm. Created in BioRender. Wang, X. (2026) https://BioRender.com/rvdsq5t. (**C**) Bacterial growth in Col-0 leaves at 3 dpi. Data are pooled from three independent experiments and shown as mean ± SD (mock, *n* = 35; 6-OHNA, *n* = 34; SA, *n* = 34); *P* values were calculated using unpaired two-tailed Student’s *t* tests. (**D**) Schematic overview of SA- and NHP-mediated immune pathways in *Arabidopsis*. (**E** and **F**) 6-OHNA induces expression of immune-related genes and promotes accumulation of defense-associated metabolites, including SA, SA glucoside (SAG), and pipecolic acid (Pip). Transcript levels were quantified by reverse transcription quantitative polymerase chain reaction [RT-qPCR; (E)], and metabolites (F) were measured 24 hours after treatment. Data are mean ± SD (*n* = 9). Two-tailed paired Student’s *t* tests were used. (**G** to **J**) SA biosynthesis and downstream NPR1 signaling are both required for 6-OHNA–induced resistance. Disease symptoms [(G) and (I)] and bacterial growth quantification [(H) and (J)] in the SA-deficient mutant *sid2-2* and SA signaling mutant *npr1-2*, respectively, following mock, 6-OHNA, or SA treatment. Data are mean ± SD. Sample sizes: *sid2-2* (mock, *n* = 34; 6-OHNA, *n* = 34; SA, *n* = 35); *npr1-2* (mock, *n* = 34; 6-OHNA, *n* = 33; SA, *n* = 34). Two-tailed unpaired Student’s *t* tests were used. Scale bars, 1 cm.

Having established that specific root-associated bacteria convert host-derived VB_3_ into 6-OHNA, we next investigated whether this microbial metabolite exerts biological effects on the host plant. Given the structural resemblance of 6-OHNA to salicylic acid (SA) and *N*-hydroxy pipecolic acid (NHP), two central defense signaling molecules that orchestrate local and systemic immunity in plants (*40*), we hypothesized that 6-OHNA may likewise function as an immune signal. Because recognition by the SA receptors NPR1 and NPR4 is central to SA-mediated immunity, we first tested whether 6-OHNA is recognized by these receptors, both of which bind SA with high affinity (*41*, *42*). Binding assays revealed that 6-OHNA interacted with both NPR1 and NPR4. However, its binding affinity was substantially weaker than that of SA, with a dissociation constant (*K*_d_) of 7.19 ± 1.74 μM for NPR1 (*n* = 3; Fig. 4A) and 1.35 ± 0.42 μM for NPR4 (*n* = 3; fig. S10), compared with the reported SA affinities of ∼220 nM for NPR1 and 20 to 50 nM for NPR4 (*41*, *42*). These markedly reduced affinities suggest that direct recognition by NPR proteins is unlikely to account for the biological activity of 6-OHNA in planta.

We therefore considered the alternative possibility that 6-OHNA promotes immunity indirectly by stimulating endogenous SA biosynthesis and subsequent NPR1-dependent signaling. To test this hypothesis, we pretreated plants with 6-OHNA before challenge with *Pseudomonas syringae* pv. tomato DC3000 (*Pst* DC3000). Compared with mock treatment, 6-OHNA pretreatment significantly enhanced resistance, as evidenced by attenuated disease symptoms and reduced bacterial titers, although the effect of 6-OHNA was weaker than that of SA (Fig. 4, B and C). 6-OHNA did not affect bacterial growth in vitro (fig. S11), indicating that the enhanced resistance resulted from the activation of host immunity rather than direct antimicrobial activity.

Transcriptome-guided analyses revealed that 6-OHNA triggered canonical defense responses in the absence of pathogen challenge. Key SA-responsive genes (*PR1*, *PR2*, and *PR5*) and SA biosynthetic genes (*ICS1*, *EDS5*, and *PBS3*) were significantly up-regulated (*40*, *43*), with the expression of *PR1* and *ICS1* increasing by ∼5.0- and 1.8-fold, respectively, compared with that of the much stronger induction by SA (385- and 3.2-fold, respectively) (Fig. 4, D and E). In parallel, core components of the NHP pathway (*ALD1*, *SARD4*, and *FMO1*) were transcriptionally activated (*44*–*46*), with *ALD1* and *FMO1* induced by 4.0- and 5.8-fold, respectively (Fig. 4, D and E).

Consistent with these transcriptional changes, targeted metabolite profiling revealed increased accumulation of SA, its conjugate SA glucoside (SAG), and the NHP precursor pipecolic acid (Pip), with SA and Pip levels elevated by approximately 2- to 4-fold and 1.5- to 4-fold, respectively (Fig. 4F). These data indicate that 6-OHNA alone is sufficient to coordinately activate SA- and NHP-associated immune networks. Notably, SA treatment itself induced Pip accumulation and NHP-related gene expression, reinforcing the existence of a tightly coupled SA-NHP signaling axis.

To define the genetic requirements underlying this response, we examined the SA-deficient mutant *sid2-2* and the SA receptor mutant *npr1-2*. In contrast to wild-type plants, both *sid2-2* and *npr1-2* failed to acquire 6-OHNA–induced resistance and displayed disease severity and pathogen titers that were indistinguishable from those of mock-treated controls (Fig. 4, B, C, and G to J). This loss of resistance was accompanied by the complete absence of SA- and NHP-associated transcriptional and metabolic activation (fig. S12). Notably, exogenous SA fully restored disease resistance in *sid2-2* but not in *npr1-2*, confirming that SA biosynthesis induction and SA receptor NPR1–mediated signaling are indispensable for 6-OHNA–triggered immune responses (Fig. 4, G to J, and fig. S12). Together, these results demonstrate that 6-OHNA–triggered immune priming requires both endogenous SA biosynthesis and NPR1-dependent signaling. Thus, 6-OHNA functions as a microbe-derived signal that activates plant immunity through the SA-NPR1 signaling pathway and its associated SA-NHP defense network.

### Root bacterial 6-OHNA is a systemically mobile signal that primes plant immunity

To determine whether microbially produced 6-OHNA activates plant immunity in planta, we colonized Col-0 seedlings with the rhizobacterium *Ensifer* A311 (Fig. 3C). Compared with noninoculated control plants, colonized plants presented significantly fewer disease symptoms and lower *Pst* DC3000 titers. Consistently, targeted metabolite profiling revealed pronounced accumulation of 6-OHNA in both the roots and leaves of *Ensifer* A311–colonized plants. These findings support a model in which root bacteria–derived 6-OHNA contributes to enhanced disease resistance in planta (fig. S13).

**Fig. 5.**
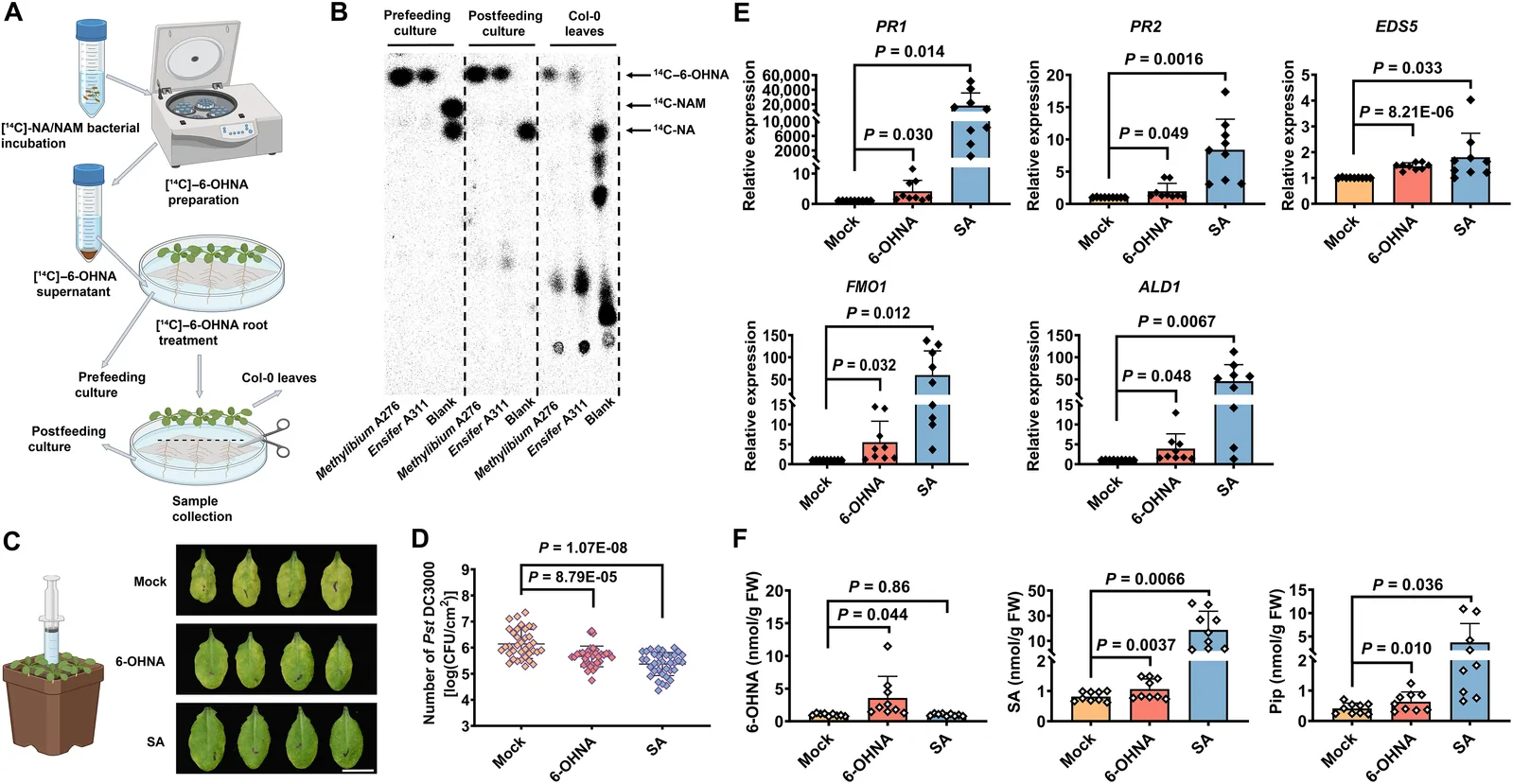
6-OHNA mediates long-distance immune priming in *Arabidopsis*. (**A**) Experimental workflow for the production and application of radiolabeled 6-OHNA. [^14^C]–6-OHNA was generated by bacterial conversion of [^14^C]-NA or NAM and applied to the roots of Col-0 seedlings. Bacterial supernatants before and after plant feeding, as well as leaf tissues, were collected for radiotracer analysis. Created in BioRender. Wang, X. (2026) https://BioRender.com/x9us04j. (**B**) Radiotracer analysis of 6-OHNA translocation. [^14^C]–6-OHNA produced by *Methylibium* A276 or *Ensifer* A311 was applied to Col-0 roots. Radio-TLC detected [^14^C]–6-OHNA in bacterial supernatants before (prefeeding) and after (postfeeding) coculture, as well as in distal leaves. Blank: half-strength tryptone soya broth (½ TSB) medium. (**C**) Root-applied 6-OHNA enhances systemic resistance to *Pst* DC3000. Disease symptoms in distal leaves were assessed at 3 dpi following root treatment with 6-OHNA, SA, or mock. Representative images from three independent experiments are shown. Scale bar, 1 cm. Created in BioRender. Wang, X. (2026) https://BioRender.com/iav1h4m. (**D**) Bacterial growth in systemic leaves at 3 dpi following root treatment. Data are pooled from three independent experiments and shown as mean ± SD (mock, *n* = 36; 6-OHNA, *n* = 35; SA, *n* = 36). *P* values were calculated using unpaired two-tailed Student’s *t* tests. (**E**) Root-applied 6-OHNA induces systemic expression of systemic acquired resistance marker genes. Transcript levels of *PR1*, *PR2*, *EDS5*, *ALD1*, and *FMO1* in distal leaves were quantified by RT-qPCR. Data represent mean ± SD (*n* = 9). Statistical significance was assessed by two-tailed paired Student’s *t* tests. (**F**) Accumulation of immune-associated metabolites in distal leaves following root treatment. Levels of 6-OHNA, SA, and Pip were quantified 24 hours after treatment in the same samples as in (E). Data are mean ± SD (*n* = 9). Two-tailed paired Student’s *t* tests were used.

Given the relatively modest immune activation elicited by exogenous 6-OHNA, we next investigated whether root bacteria–derived 6-OHNA functions as a long-distance immune signal. To directly assess its mobility, we traced [^14^C]-labeled 6-OHNA and observed clear root-to-shoot translocation, providing direct evidence that 6-OHNA is a systemically mobile metabolite capable of long-distance transport from roots to aerial tissues (Fig. 5, A and B). Consistent with these observations, the application of unlabeled (cold) 6-OHNA to roots resulted in dose-dependent accumulation in distal leaves within 2 days, independently confirming long-distance transport under physiological conditions (fig. S14).

We then tested whether this mobility is coupled to systemic immunity. Root application of 6-OHNA enhanced resistance in distal leaves, manifested as reduced disease symptoms and suppressed pathogen growth, phenocopying SA treatment but with weaker efficacy (Fig. 5, C and D). In line with this protection, systemic leaves showed the induction of canonical defense genes and the accumulation of defense-associated metabolites, including 6-OHNA itself, SA, and Pip (Fig. 5, E and F). Together, these results identify 6-OHNA as a microbially derived, systemically mobile metabolite that primes SA-dependent immune responses in distal tissues, thereby enhancing resistance to aerial pathogens.

### Bacterial *nicAB* and plant-derived VB_3_ enable plant immune priming

**Fig. 6.**
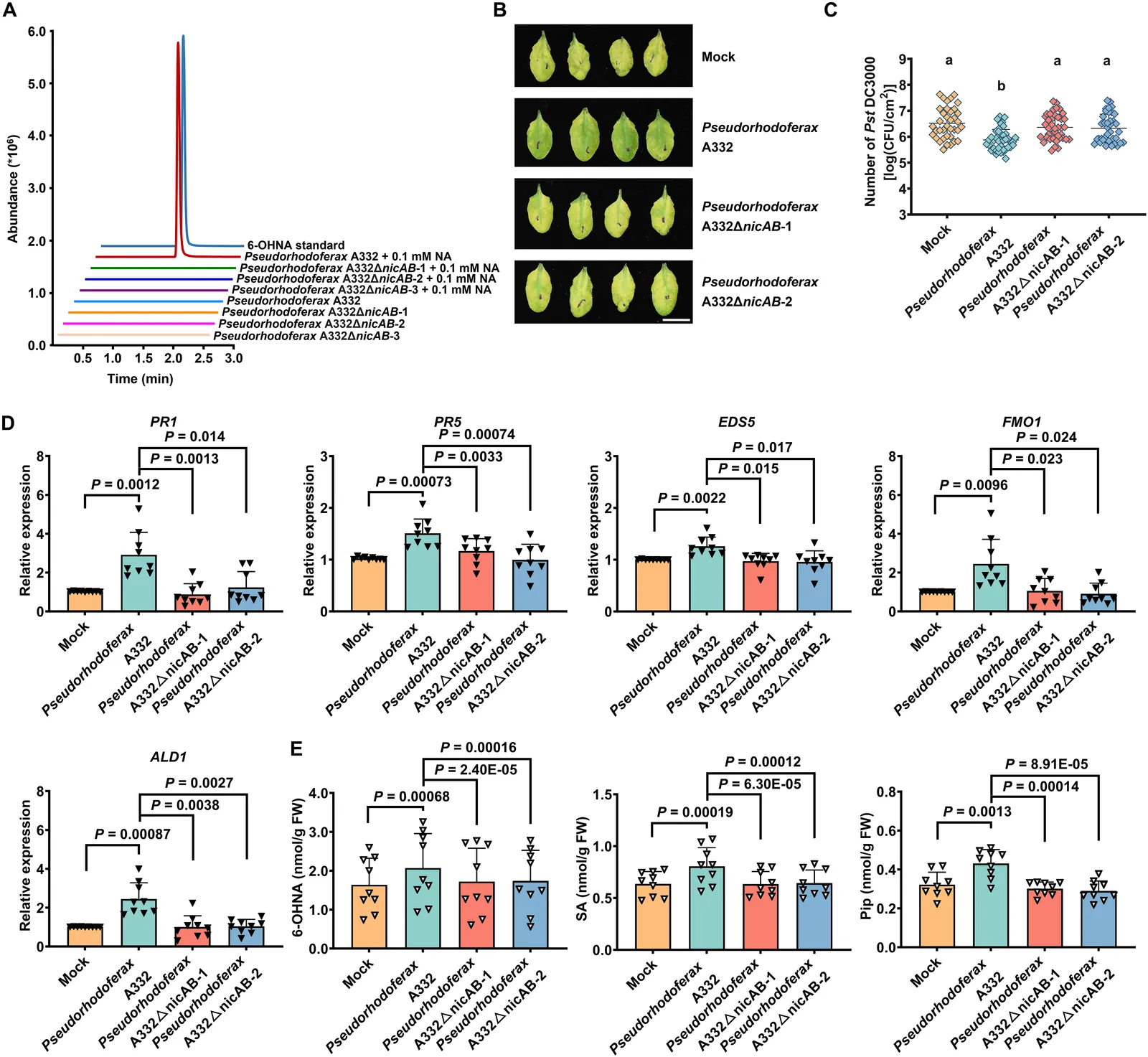
A *nicAB*-dependent root bacterial pathway fueled by plant-derived VB_3_ activates systemic immunity in *Arabidopsis*. (**A**) 6-OHNA production by wild-type *Pseudorhodoferax* A332 and *nicAB* knockout mutants. Bacteria were cultured with or without 100 μM NA for 8 hours, and 6-OHNA in culture supernatants was quantified by LC-QQQ-MS. (**B**) Systemic disease symptoms in Col-0 following root colonization by wild-type *Pseudorhodoferax* A332, *nicAB* mutants, or mock treatment. The seventh to ninth leaves were inoculated with *Pst* DC3000 and photographed at 3 dpi. Representative images from three independent experiments are shown. Scale bar, 1 cm. (**C**) Bacterial growth in systemic leaves at 3 dpi. Data are pooled from three independent experiments and shown as mean ± SD (mock, *n* = 37; *Pseudorhodoferax* A332, *n* = 41; A332Δ*nicAB*-1, *n* = 40; A332Δ*nicAB*-2, *n* = 37). Statistical significance was assessed by one-way analysis of variance (ANOVA) with Tukey’s multiple-comparison test; different letters indicate significant differences (*P* < 0.05). (**D**) Root colonization by wild-type A332 induces systemic expression of immune-related genes. Transcript levels of *PR1*, *PR5*, *EDS5*, *FMO1*, and *ALD1* in systemic leaves were quantified by RT-qPCR. Data represent mean ± SD (*n* = 9). Statistical significance was evaluated using two-tailed paired Student’s *t* tests. (**E**) Accumulation of immune-associated metabolites in systemic leaves following root colonization. Levels of 6-OHNA, SA, and Pip were quantified 24 hours after treatment in the same samples as in (D). Data are mean ± SD (*n* = 9). Two-tailed paired Student’s *t* tests were used.

If 6-OHNA functions as a biologically relevant microbial signal, then bacterial production of 6-OHNA should contribute to microbe-mediated disease resistance. We therefore selected *Pseudorhodoferax* A332, a root-derived isolate that secretes 6-OHNA and is genetically tractable, as *Ensifer* A311 is not amenable to genetic manipulation (Fig. 3C and figs. S15 and S16). Deletion of *nicAB* completely abolished 6-OHNA production and impaired bacterial growth on NA or NAM, demonstrating that *nicAB* is essential for NA metabolism in this strain (Fig. 6A and fig. S17). We next examined the immunological consequences of *nicAB* loss in planta. Root inoculation with wild-type A332 conferred mild systemic resistance to *Pst* DC3000, whereas *nicAB*-deficient mutants failed to do so (Fig. 6, B and C). To determine whether this loss of immune-inducing capacity was due to impaired colonization, we quantified bacterial abundance and observed no significant difference in root colonization between wild-type A332 and the Δ*nicAB* mutant under our experimental conditions (fig. S18). Consistent with this, only wild-type A332 but not the Δ*nicAB* mutants induced modest but significant up-regulation of defense-associated genes, including *PR1*, *PR5*, *EDS5*, *FMO1*, and *ALD1* (Fig. 6D). This immune activation was accompanied by the coordinated accumulation of 6-OHNA, SA, and Pip in systemic leaves (Fig. 6E). Together, these findings identify *nicAB* as a conserved bacterial determinant of 6-OHNA biosynthesis and demonstrate that microbially derived 6-OHNA contributes to the priming of systemic immunity in *Arabidopsis*.

**Fig. 7.**
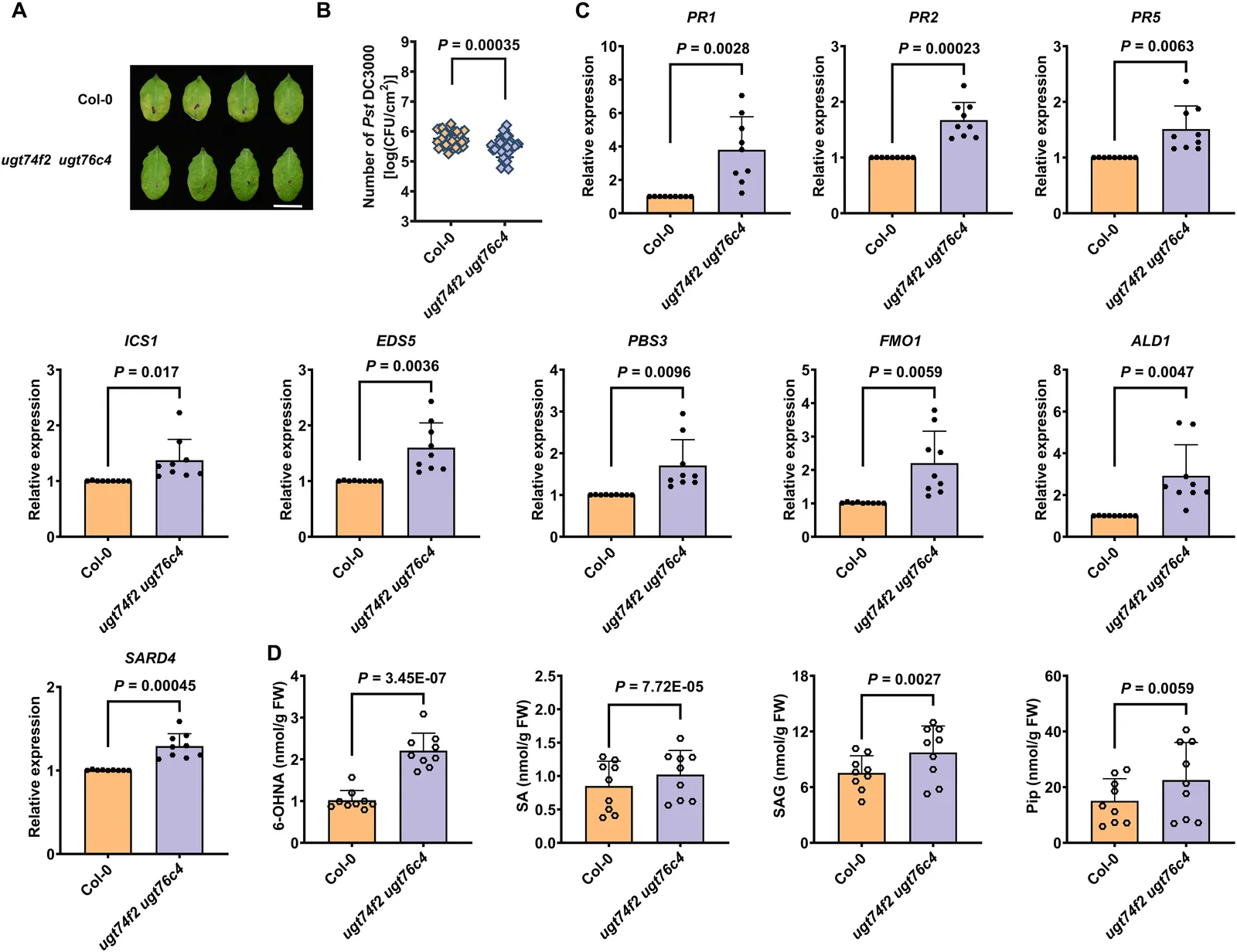
Enhanced systemic immune activation and disease resistance in natural soil–grown VB_3_-oversecreting *ugt74f2 ugt76c4* plants. (**A**) Enhanced disease resistance in soil-grown *ugt74f2 ugt76c4* plants. Disease symptoms on the seventh to ninth leaves of 5-week-old Col-0 and *ugt74f2 ugt76c4* plants were assessed at 3 dpi with *Pst* DC3000. Representative images from three independent experiments are shown. Scale bar, 1 cm. (**B**) Bacterial growth in Col-0 and *ugt74f2 ugt76c4* leaves at 3 dpi. Data are mean ± SD (*n* = 9). Two-tailed paired Student’s *t* tests were used. (**C**) Basal expression of immune-related genes in systemic leaves of soil-grown plants without pathogen challenge. Data represent mean ± SD (*n* = 9). Two-tailed paired Student’s *t* tests were used. (**D**) Basal accumulation of immune-related metabolites in systemic leaves of soil-grown plants. Levels of 6-OHNA, SA, SAG, and Pip were quantified in the same samples as in (C). Data are mean ± SD (*n* = 9). Two-tailed paired Student’s *t* tests were used.

In addition to bacterial *nicAB*, we investigated the contribution of plant-derived VB_3_ to this immune-modulatory system. To assess the ecological relevance of host-secreted VB_3_ as a precursor for microbial 6-OHNA production, we grew the VB_3_-oversecreting *ugt74f2 ugt76c4* mutant and Col-0 in natural soil. Notably, *nic1-1*, which secretes the largest amount of VB_3_ (Fig. 1B), was excluded because its altered NAD(P)(H) homeostasis and heightened sensitivity to abscisic acid (ABA) and salt (*10*) would complicate interpretation in microbiome-mediated 6-OHNA experiments. Upon challenge with *Pst* DC3000, *ugt74f2 ugt76c4* plants exhibited markedly enhanced disease resistance relative to Col-0, as evidenced by reduced disease symptoms and decreased pathogen proliferation (Fig. 7, A and B). This improved resistance was associated with constitutive immune priming, characterized by elevated expression of pathogenesis-related genes, core components of SA biosynthesis, and key regulators of the NHP pathway. Consistent with this transcriptional activation, the systemic leaves of *ugt74f2 ugt76c4* plants accumulated higher levels of immune-related metabolites, including 6-OHNA, SA and its conjugate, and Pip (Fig. 7, C and D).

**Fig. 8.**
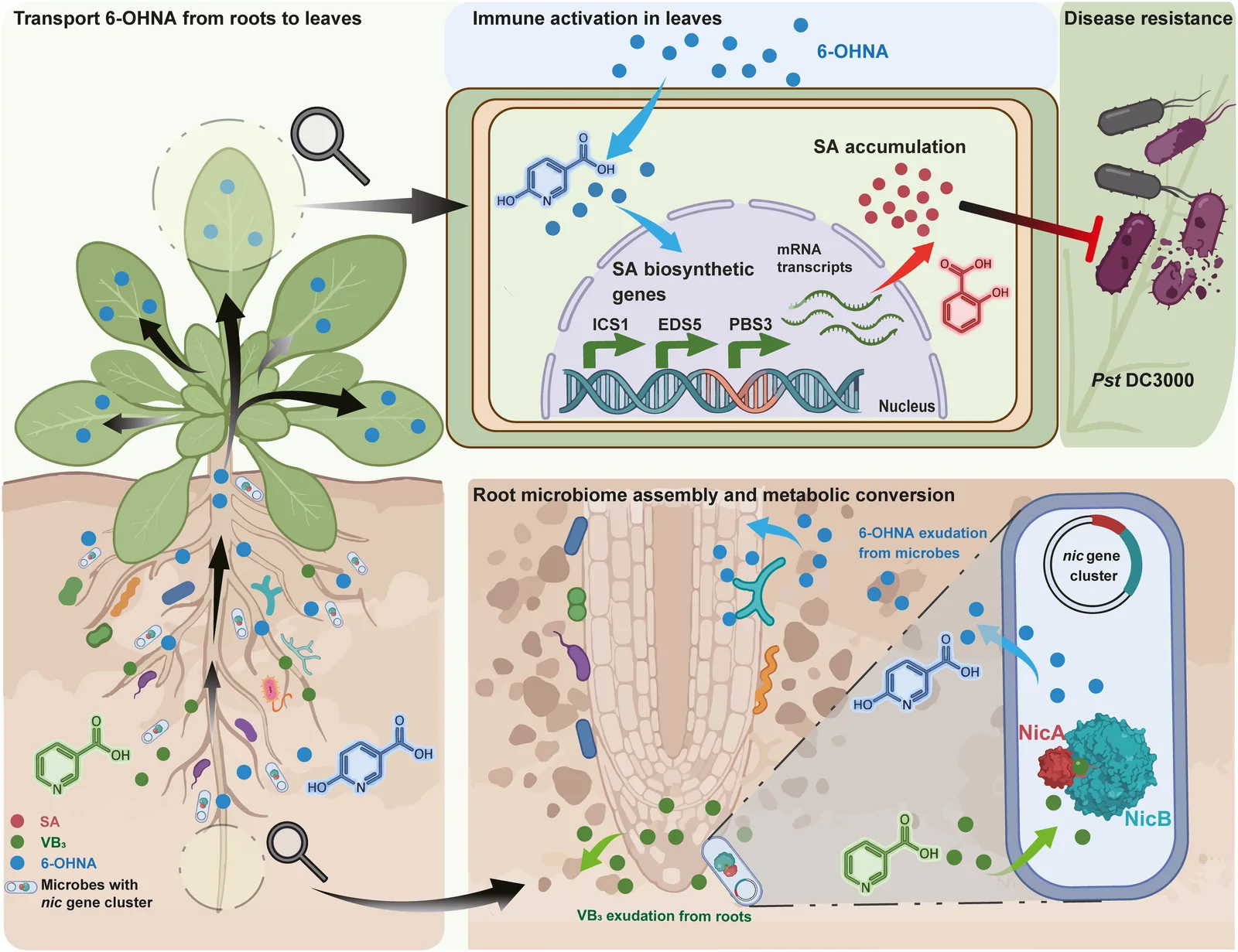
Model of rhizobacteria-derived 6-OHNA in plant immune regulation. Host-secreted VB_3_ is taken up by rhizosphere bacteria carrying functional *nic* BGCs and converted into 6-OHNA via the nicAB enzymatic complex. Microbially produced 6-OHNA acts as a mobile signal that promotes bacterial growth and systemically primes SA-dependent immunity in *Arabidopsis thaliana*. In distal leaves, 6-OHNA induces the expression of core SA biosynthetic genes (*ICS1*, *EDS5*, and *PBS3*), leading to increased SA accumulation and activation of immune priming. This enhances resistance against the foliar pathogen *Pst* DC3000. Collectively, this metabolic exchange links plant-derived nutrients, rhizobacterial metabolism, and systemic immune regulation to shape disease resistance.

Together, these findings demonstrate that the native root microbiota efficiently converts increased host VB_3_ secretion into 6-OHNA, which is sufficient to prime systemic immunity even in the absence of pathogen challenge. Combined with our bacterial genetic analyses, these results identify 6-OHNA as a key microbial signal underlying systemic immunity induced by root bacteria. We propose a model in which host-derived VB_3_ functions as both a nutrient and an ecological cue, selectively enriching *nic* BGC–harboring bacteria with immunomodulatory capacity and thereby linking root microbiota composition to SA-dependent systemic defense activation (Fig. 8).

## DISCUSSION

Our study reveals a previously unrecognized metabolic dialog between plants and their root microbiota. We show that plant-derived VB_3_ not only shapes root microbiome assembly but is also further transformed by specialized bacterial taxa into 6-OHNA, a microbially derived metabolite that primes systemic immunity in *Arabidopsis*. These findings expand the current understanding of rhizosphere chemical communication by demonstrating how host metabolites can be metabolically remodeled by associated microbes to generate bioactive compounds with direct consequences for plant defense.

Recent advances have established that rhizosphere metabolites are not passive exudates but are dynamic mediators of plant-microbe interactions. Plants continuously secrete structurally diverse small molecules into the rhizosphere, where microbial metabolism further expands the chemical landscape and generates biologically active derivatives. Several bacterial taxa have been shown to activate plant immunity; for example, *Clostridium* spp. increase rice resilience through immune stimulation (*47*), and *Bacillus cereus* NJ01 triggers EDS1-WRKY18–mediated, SA- and ABA-dependent immunity in *Arabidopsis* (*48*). Our identification of 6-OHNA as a root bacteria-derived immunomodulator adds a previously unrecognized dimension to this chemical dialog. By inducing a canonical SA-dependent transcriptional program and metabolic rewiring in the absence of pathogens, 6-OHNA functions as a metabolic priming cue that sensitizes plants to heightened immune responsiveness.

Although 6-OHNA was capable of binding NPR1 and NPR4, its affinity for these receptors was substantially weaker than that of SA (Fig. 4A and fig. S10), suggesting that 6-OHNA is unlikely to function simply as a direct SA mimic. Instead, our genetic and biochemical analyses support a model in which 6-OHNA promotes endogenous SA biosynthesis and subsequently activates NPR1-dependent signaling to establish systemic immunity (*41*). 6-OHNA is detectable endogenously in *Arabidopsis* tissues. These findings raise fundamental questions regarding its prevalence and spatial distribution within plants, the extent to which its biosynthetic route in plants resembles or diverges from that of microbial pathways (*20*, *21*), and whether endogenous 6-OHNA performs additional physiological functions beyond immune priming.

Despite these mechanistic insights, the ecological and evolutionary scope of this mechanism, however, remains unresolved. Although plant secretion of VB_3_ is widespread, bacteria harboring *nic* BGCs are abundant, accounting for 16.6% of the cultured root isolates in this study and are likely present at comparable frequencies in public soil microbiome datasets (*39*). However, the environmental distribution and abundance of 6-OHNA in natural rhizospheres remain unknown. Consequently, it is still premature to conclude that 6-OHNA–mediated immune priming represents a conserved strategy across plant species and ecosystems. Future studies integrating quantitative metabolite profiling of natural soils, comparative genomics of *nicAB*-type BGCs, and functional analyses across diverse host species will be necessary to determine whether 6-OHNA functions as a broadly conserved rhizosphere signal or instead acts as a context-dependent immunomodulator restricted to specific plant-microbe associations.

As illustrated in the working model (Fig. 8), *nic*-containing bacteria face a resource allocation trade-off: fully catabolizing 6-OHNA for their own growth versus excreting a fraction to “pay” the plant for immune priming. In competitive rhizosphere environments, this apparent metabolic cost can be offset if plant-mediated benefits, such as increased root exudation, reduced pathogen pressure, or improved niche stability, and feedback support bacterial fitness. Under such conditions, reciprocal exchange between the host and microbiota may result in a mutually beneficial interactions, in which bacterial investment in 6-OHNA production promotes host defense, while the host, in turn, sustains a favorable niche for these microbes. This positive feedback loop provides a plausible basis for the evolutionary stabilization of this trait and suggests that 6-OHNA–mediated signaling could represent an emergent outcome of plant-microbe coevolution rather than a unidirectional metabolic byproduct (*39*, *49*, *50*).

Together, our findings demonstrate that the microbial metabolism of plant-derived nutrients can generate signaling molecules that directly shape host immune phenotypes. This work reveals an additional layer of rhizosphere chemical communication that links metabolic cooperation between plants and microbiota to immune outcomes, thereby broadening our understanding of how beneficial microbial functions emerge within complex root-associated communities.

## MATERIALS AND METHODS

### Plant materials

*A. thaliana* accession Col-0 was used as the wild type in this study. All mutant lines were in the Col-0 background. The following mutants were used, with gene locus identifiers and allele names indicated in parentheses: *nic1-1* (*AT2G22570*, SALK_131410), *ugt74f2* (*AT2G43820*, EMS line), *ugt74f2 ugt76c4* (*AT2G43820*/*At5G05880*, CS25037), *sid2-2* (*AT1G74710*, SALK_088254), and *npr1-2* (*AT1G64280*, EMS line). Homozygous lines were confirmed by polymerase chain reaction (PCR)–based genotyping, and mutations were further verified by Sanger sequencing using primers listed in table S3.

### Growth and harvesting of *A. thaliana* in natural soil

Seeds of Col-0, *nic1-1*, *ugt74f2*, and *ugt74f2 ugt76c4* were surface sterilized and grown in natural soil collected from Changping Farm, Beijing, China (40.109°N, 116.424°E). Plants were cultivated under controlled short-day conditions (10-hour light/14-hour dark, 22°C, and 50% relative humidity), as described previously (*37*).

For root microbiome profiling, plants were grown for 6 weeks. Roots were carefully harvested and washed three times in phosphate-buffered saline (PBS; pH 7.4) with gentle shaking (180 rpm and 15 min per wash) to remove adhering soil particles and loosely associated microorganisms. Washed roots, together with unplanted bulk soil samples, were flash frozen in liquid nitrogen and stored at −80°C until further processing. For disease resistance assays and immune priming analyses, Col-0 and *ugt74f2 ugt76c4* plants were grown for 5 weeks in the same natural soil before pathogen inoculation or tissue collection for molecular analyses.

### DNA isolation and construction of 16*S* rRNA gene libraries

Two-step PCR amplicon library preparation was performed as described previously (*31*) with minor modifications. Total DNA was extracted from frozen root and bulk soil samples using the FastDNA Spin Kit (MP Biomedicals), following the manufacturer’s instructions. DNA concentrations were quantified using the PicoGreen dsDNA Assay Kit (Thermo Fisher Scientific), and all samples were normalized to 3.5 ng μl^−1^ before amplification.

The V5-to-V7 hypervariable region of the bacterial 16*S* rRNA gene was amplified using barcoded primers 799F and 1193R. Each 30 μl of PCR reaction contained 10 ng of template DNA, 0.25 μM each primer, 0.2 mM deoxynucleotide triphosphates, 1 mM MgCl_2_, 0.75 U of PrimeSTAR HS DNA polymerase (Takara), and 1× reaction buffer. PCR was performed under the following conditions: initial denaturation at 98°C for 30 s; 25 cycles of 98°C for 10 s, 55°C for 15 s, and 72°C for 1 min; and a final extension at 72°C for 5 min. All reactions were performed in triplicate for each sample.

Triplicate PCR products were pooled and size selected by electrophoresis on 1.2% agarose gels (Lonza). Target bands were excised and purified using the Wizard SV Gel and PCR Clean-Up System (Promega), followed by further purification with AMPure XP magnetic beads (Beckman Coulter). Final libraries were subjected to paired-end sequencing on an Illumina HiSeq 2500 platform.

### Microbiota analysis

Microbiota analysis was performed using a pipeline adapted from (*31*). Raw 16*S* rRNA gene sequences were processed using QIIME v1.9.1 (*51*), USEARCH v10.0 (*52*), and in-house scripts. Paired-end Illumina reads were checked by FastQC and merged using join_paired_ends.py. Demultiplexing was performed by barcode extraction (extract_barcodes.py) followed by sample assignment of reads with split_libraries_fastq.py from QIIME. Amplicon primers were trimmed using Cutadapt v1.14 (*53*).

For ASV clustering, redundant sequences were removed via the -fastx_uniques command in USEARCH. Chimera filtering and sequence denoising were performed with the -unoise3 algorithm. An ASV table was generated using -otutab. The representative sequences were then taxonomically classified using the RDP classifier (*54*).

Differential abundance analysis was performed with the edgeR package (*55*) using a negative binomial generalized linear model. ASVs with a fold change > 1.2 and a false discovery rate (FDR) < 0.2 were deemed statistically significant. Comparisons were conducted between Col-0 and each NA-related mutant line (*nic1-1*, *ugt74f2*, and *ugt74f2 ugt76c4*). Overlapping ASVs across comparisons were visualized using the VennDiagram package in R (*56*). All data visualization was performed using ggplot2 unless otherwise stated (*57*).

### Isolation and cultivation of root-associated bacteria

Root-associated bacterial isolates were obtained from *A. thaliana* Col-0 plants grown in natural soil collected from Changping Farm, Beijing, China, as part of a previously established *A. thaliana* root microbiome culture collection. Bacteria were isolated by serial dilution plating on solid medium. Resulting colonies were initially identified by barcoded 16*S* rRNA gene sequencing, and isolates sharing >97% sequence identity with reference sequences in the 16*S* rRNA gene database were selected for further purification.

Individual colonies were streaked three times for purification on half-strength tryptone soya broth (½ TSB) agar plates [TSB (15 g liter^−1^) and agar (20 g liter^−1^); Oxoid] and incubated at 28°C overnight. A single colony from each purified strain was subsequently inoculated into liquid ½ TSB medium and cultured at 28°C with shaking at 220 rpm for ∼48 hours.

### Genomic DNA extraction, whole-genome sequencing, and de novo assembly of bacterial isolates

The procedures for genomic DNA extraction, whole-genome sequencing, and de novo assembly were performed as previously described (*39*). Briefly, genomic DNA was extracted from 500 μl of bacterial cultures. Cell lysis was performed by mechanical homogenization in Lysing Matrix E tubes using a Precellys Evolution homogenizer (Bertin Technologies), with 2 cycles of 30 s at 7200 rpm. DNA was purified using a FastDNA Spin kit (MP Biomedicals) following the manufacturer’s instructions. DNA concentrations were measured with a PicoGreen dsDNA Assay kit (Life Technologies), and 1 μg of DNA per strain was used for library preparation.

Whole-genome sequencing libraries were sequenced on an Illumina NovaSeq 6000 platform to generate 150–base pair (bp) paired-end reads, targeting an average sequencing depth of approximately 20× per genome, corresponding to ∼1 Gbp of raw data per strain. Adapter trimming and quality filtering were performed using Trimmomatic v0.39 with parameters “SLIDINGWINDOW:4:20” and “MINLEN:100.” Cleaned reads were assembled de novo with SPAdes v3.14.0 using options “–isolate, –careful, –cov-cutoff auto.” Contigs shorter than 1000 bp were excluded from downstream analyses.

Assembly quality was assessed using the CheckM v1.1.8 lineage_wf workflow to estimate completeness and contamination. Draft genomes with >5% contamination were processed through the MetaWRAP v1.3.2 binning module to resolve mixed cultures into separate, pure genomic bins. This pipeline yielded a total of 441 unique bacterial genomes from *A. thaliana* roots, all with contamination below 5.0%. Most genomes met high-quality draft standards, exhibiting >90.0% completeness while maintaining contamination below the 5.0% threshold.

### Correlation analysis between ASVs and metabolites

Pairwise Spearman rank correlations were calculated between the mean relative abundances of 637 differentially enriched ASVs (abundance > 0.005% and occurrence frequency > 30% across root microbiota samples) and the concentrations of five NA-related metabolites (VB_3_, NA*O*G, NA*N*G, NAD, and Tg). Correlation analyses were performed on genotype-level mean. Owing to the limited number of observations (*n* = 4), formal statistical inference was not applied; correlations were treated as descriptive, with an effect-size threshold of |ρ| ≥ 0.8 used to define robust associations. Spearman correlations were computed using the R package psych (v2.4.12) (*58*), and heatmaps were visualized using the R package ComplexHeatmap (v2.22.0) (*59*). ASVs were ordered by phylum-level taxonomy (*Pseudomonadota* are shown at class level). The VB_3_-positive high-correlation ASVs (VB_3_ ρ ≥ 0.8; *n* = 60) were highlighted with a red sidebar annotation.

For network visualization, ASVs with VB3 ρ ≥ 0.8 (*n* = 60) were displayed in a three-layer radial network graph constructed using the R package ggplot2 (v4.0.2) (*57*). The central node represents the VB_3_ metabolite; the second layer represents phylum/class-level taxonomy nodes; and the outer layer represents individual ASV nodes. ASV nodes were colored by phylum, with *Pseudomonadota* shown at the class level. ASVs were linked to corresponding culturable isolates from an *Arabidopsis* root microbiota collection based on >97% 16*S* rRNA gene sequence identity.

### β-Diversity and pairwise group comparisons

To assess differences in root bacterial community composition among four genotypes (Col-0, *nic1-1*, *ugt74f2*, and *ugt74f2 ugt76c4*), β-diversity was calculated using Bray-Curtis distance on ASV reads table. Constrained principal coordinate analysis (CPCoA) was performed using the R package vegan (v2.6-8) (*60*) with genotype as the constraining variable. The overall significance of the genotype effect was assessed by permutational multivariate analysis of variance (PERMANOVA).

Pairwise differences between all genotype comparisons were tested using PERMANOVA with Bray-Curtis distance. *P* values from pairwise comparisons were corrected for multiple testing using the Benjamini-Hochberg FDR method. Adjusted *P* values are visualized as a symmetric matrix heatmap.

### Metabolic analysis of [^14^C]-NA or [^14^C]-NAM in *Arabidopsis* root bacteria

Fresh cultures of *Arabidopsis* root bacterial isolates were streaked onto ½ TSB agar plates [TSB (15 g liter^−1^) and agar (20 g liter^−1^)] and incubated at 28°C overnight. Single colonies were inoculated into 4 ml of liquid ½ TSB medium and cultured at 28°C for 48 hours. Subsequently, 1 ml of aliquots of each culture were supplemented with 1 μl of 100 μM [^14^C]-NA or [^14^C]-NAM and incubated for an additional 8 hours.

Cells were harvested by centrifugation at 20,000*g* for 10 min. Supernatants were transferred to fresh tubes, and cell pellets were washed twice with 0.5 ml of resuspension buffer [10 mM tris-HCl (pH 7.4), 150 mM NaCl, and 1 mM MgCl_2_] before being resuspended in 300 μl of the same buffer.

To achieve complete cell lysis while preserving radiolabel integrity, samples were subjected to 5 cycles of freezing in liquid nitrogen (8 min), thawing in an ice-water bath (10 min), and ultrasonication for 10 min in an ice-water bath using a water-bath sonicator.

Radiolabeled metabolites were separated by thin-layer chromatography (TLC) as described previously (*10*), with minor modifications. Aliquots (15 μl) of culture supernatants and cell lysates were spotted onto Polygram TLC plates (Macherey-Nagel) and developed using *n*-butanol:acetic acid:water [2:1:1 (v/v/v)] as the mobile phase. Authentic chemical standards were cospotted for metabolite identification. Radioactive signals were detected and quantified using an InstantImager radio scanner (Packard Instrument Company).

### BGC analysis

BGCs were predicted from bacterial isolate genomes using antiSMASH v7.1.0 (*61*) with default parameters, enabling the detection of known clusters, subclusters, and general features (--cb-knownclusters, --cb-subclusters, and --cb-general). To reduce potential bias arising from fragmented genome assemblies, only BGCs located on contigs longer than 5 kb were retained for downstream analyses. Identified BGCs were classified into major biosynthetic categories, including terpene, NRPSs, PKSs, RiPPs, and others.

### Identification and comparative analysis of *nic* BGCs

The genomes of all 22 VB_3_-associated isolates were screened for the presence of *nic* BGC. Open reading frames within these regions were annotated using the Prokka pipeline, and gene functions were assigned on the basis of sequence homology to previously characterized *nic* genes (*20*).

For comparative genomic analyses, *nic* BGCs were aligned and visualized in R (v4.4.1) using gggenes and ggplot2 packages. The reference strain, *P. putida* KT2440 (*20*), was included for comparison.

### Phylogenetic classification and tree construction

All bacterial genomes were taxonomically classified using GTDB-Tk v2.0.0 with GTDB release R207. A maximum-likelihood phylogenetic tree was constructed de novo using the *infer* workflow in GTDB-Tk v2.0.0 (*62*), which is based on a concatenated alignment of 120 conserved bacterial marker genes. Amino acid sequence alignments were generated using the WAG substitution model. Taxonomic ranks from class to genus were assigned according to GTDB taxonomy and visualized alongside the phylogenetic tree using Interactive Tree Of Life (*63*).

### Bacterial growth assays using NA or NAM as the sole nutrient source

Growth on NA or NAM as the sole carbon/nitrogen source was assessed in a defined minimal medium (MC) as described (*64*) with modifications. The MC medium contained KH_2_PO_4_ (0.33 g/liter), Na_2_HPO_4_ (1.2 g/liter), NH_4_Cl (0.11 g/liter), MgSO_4_·7H_2_O (0.10 g/liter), and CaCl_2_ (0.04 g/liter), with the pH adjusted to 7.5 before sterilization. The medium was supplemented with a 1× trace element solution and a 1× vitamin mix.

The 100× trace element stock contained nitrilotriacetic acid (1.5 g/liter), MgSO_4_·7H_2_O (3.0 g/liter), MnSO_4_·2H_2_O (0.5 g/liter), NaCl (1.0 g/liter), FeSO_4_·7H_2_O (0.1 g/liter), CoSO_4_·7H_2_O (0.18 g/liter), CaCl_2_·2H_2_O (0.1 g/liter), ZnSO_4_·7H_2_O (0.18 g/liter), CuSO_4_·5H_2_O (0.01 g/liter), KAl(SO_4_)_2_·12H_2_O (0.02 g/liter), H_3_BO_3_ (0.01 g/liter), Na_2_MoO_4_·2H_2_O (0.01 g/liter), NiCl_2_·6H_2_O (0.025 g/liter), and Na_2_SeO_3_ (0.3 mg/liter); pH adjusted to 6.5. The 1000× vitamin stock contained biotin (20 mg/liter), folic acid (20 mg/liter), pyridoxine-HCl (10 mg/liter), thiamine-HCl·2H_2_O (50 mg/liter), riboflavin (50 mg/liter), calcium d-pantothenate (50 mg/liter), vitamin B_12_ (50 mg/liter), and *p*-aminobenzoic acid (50 mg/liter). For assays, the medium was supplemented with 5 mM filter-sterilized NA or NAM.

For solid growth assays, bacterial cultures pregrown in ½ TSB were harvested, washed, and resuspended in fresh MC medium to an optical density at 600 nm (OD_600_) of 0.05 and then spotted onto MC agar [0.8% (w/v)] plates. Plates were incubated at 28°C and photographed after 5 days. For liquid assays, prepared inoculum was transferred to liquid MC medium with NA or NAM and incubated at 28°C with shaking at 200 rpm; growth (OD_600_) was monitored over time. Negative controls lacking NA or NAM were included. All experiments were performed with four biological replicates and independently repeated three times.

### Vector construction for *nicAB* genes from strain *Ensifer A311*

The coding sequences of *nicA* and *nicB* from the root bacterial strain *Ensifer* A311 were amplified by PCR using gene-specific primers (table S3). PCR products were cloned into the overexpression vectors pGreen 0029 using a 2× Seamless Cloning Mix (Biomed, China).

For plant expression, the pGreen 0029 vector was digested with ApaI and SalI and ligated with *nicA*, generating the intermediate construct pGreen-311*nicA*. This plasmid was then digested with EcoRI and ligated with *nicB* to produce the final construct pGreen-311*nicA*-311*nicB*.

All recombinant plasmids were verified by Sanger sequencing. The pGreen-311*nicA*-311*nicB* construct was transformed into *Agrobacterium tumefaciens* GV3101 (pSoup-P19) competent cells for transient expression assays in *N. benthamiana*.

### Agrobacterium-mediated transient expression in *N. benthamiana*

*A. tumefaciens* GV3101 (pSoup-P19) carrying the recombinant plasmid PGreen-311nicA-311nicB was streaked on solid LB medium supplemented with kanamycin, gentamicin, and rifampicin (50 μg ml^–1^) and incubated at 28°C for 2 days. The LB medium contained tryptone (10 g/liter), yeast extract (5 g/liter), NaCl (10 g/liter), and agar (15 g/liter).

A single colony was inoculated into liquid LB medium containing the same antibiotics [kanamycin, gentamicin, and rifampicin (50 μg ml^−1^)] and cultured at 28°C for 2 days with shaking at 220 rpm. The culture was then transferred into fresh LB medium supplemented with 10 mM MES and 40 μM acetosyringone and incubated overnight at 28°C with shaking.

Bacterial cells were collected by centrifugation (4000*g* for 10 min), washed once, and resuspended in infiltration buffer (10 mM MgCl_2_ and 200 μM acetosyringone) to an OD_600_ of 0.8 to 1.0. Bacterial suspensions were further supplemented with 0, 100 μM, or 1 mM NA as indicated and coinfiltrated into the abaxial side of three fully expanded leaves of 4- to 5-week-old *N. benthamiana* plants using a 1-ml needleless syringe.

Infiltrated plants were incubated in the dark for 24 hours and subsequently transferred to long-day conditions (16-hour light/8-hour dark, 22°C, and 50% relative humidity) for an additional 2 days. Leaf tissues were harvested 3 days postinfiltration for metabolite analysis.

### Molecular cloning of *NPRs* and recombinant protein expression and purification

The coding regions of *A. thaliana NPR1* (*AT1G64280*) and *NPR4* (*AT4G19660*) were amplified with specific primers as shown in table S3 and then cloned into the pEASY-Blunt E1 Expression vector (Transgene). All constructs were transformed into *Escherichia coli* BL21^+^ cells for prokaryotic expression. The bacteria were cultured in LB medium containing ampicillin (100 μg ml^−1^) and chloramphenicol (34 μg ml^−1^) to an OD_600_ of 0.4, followed by incubation on ice for 20 min. Protein expression was induced by adding isopropyl-β-d-thiogalactopyranoside to a final concentration of 0.4 mM, and cultures were subsequently incubated at 18°C for 20 hours. Cells were harvested by centrifugation and stored at −80°C until further use.

Recombinant proteins were purified as previously described (*42*) with minor modifications. Cell pellets were resuspended in lysis buffer containing 50 mM tris-HCl (pH 7.4), 500 mM NaCl, 10% (v/v) glycerol, 20 mM imidazole, 0.1% (v/v) Triton X-100, and 1 mM phenylmethylsulfonyl fluoride and lysed by sonication. Cell debris was removed by centrifugation at 15,000*g* for 30 min at 4°C. The clarified supernatant was loaded onto a Ni–nitrilotriacetic acid (NTA) affinity column, which was subsequently washed three times with 10 ml of lysis buffer supplemented with increasing concentrations of imidazole (20, 30, and 40 mM). Bound proteins were eluted with lysis buffer containing 250 mM imidazole.

The eluted His_6_-NPR1 protein was dialyzed and concentrated three times with PBS buffer containing 0.1% Triton X-100 at 4°C. Eluted His_6_-NPR4 proteins were treated with 200 mM dithiothreitol (DTT) for 30 min on ice, followed by dialysis and concentration against PBS buffer supplemented with 2 mM DTT and 0.1% Triton X-100 at 4°C. Purified proteins were aliquoted and stored at −80°C until use. Protein purity and concentration were assessed by SDS–polyacrylamide gel electrophoresis using bovine serum albumin as a standard.

### Microscale thermophoresis binding assay

The binding affinity between NPR1 or NPR4 and 6-OHNA was determined by microscale thermophoresis (MST) as previously described (*65*) with minor modifications. NPR protein was fluorescently labeled using a His-Tag Labeling Kit RED-tris-NTA (2nd Generation; MO-L018, NanoTemper Technologies). Labeled NPR1 or NPR4 (50 nM) was incubated with a serial dilution of 6-OHNA (6 nM to 200 μM for NPR1; 3 nM to 50 μM for NPR4) in MST assay buffer consisting of PBS supplemented with 2 mM DTT and 0.1% Triton X-100. Aliquots (10 μl) of each reaction were loaded into premium capillaries (MO-K022, NanoTemper Technologies).

Measurements were performed on a Monolith NT.115 instrument (NanoTemper Technologies) at ambient temperature, using 60% excitation power and high MST power. Data from three independent experiments were analyzed using MO.Affinity Analysis software (NanoTemper Technologies).

### Pathogenic bacteria and growth conditions

*Pst* DC3000 was cultured at 28°C on King’s B (KB) agar plates supplemented with rifampicin (50 μg ml^−1^). KB medium contained proteose peptone (20 g/liter), K_2_HPO_4_ (1.5 g/liter), MgSO_4_·7H_2_O (1.0 g/liter), agar (15 g/liter), and glycerol (10 ml/liter). For leaf infection assays, bacterial cells from log-phase overnight cultures were collected by centrifugation at 4000*g*, washed three times with sterile double-distilled water (ddH_2_O), and resuspended to a final OD_600_ of 0.001.

### Assessment of plant resistance to *Pst* DC3000

Disease resistance assays were performed as described previously (*44*, *66*) with minor modifications. Unless otherwise stated, 4- to 5-week-old *Arabidopsis* plants were used.

For chemical-induced resistance assays, plants were pretreated with 1 mM 6-OHNA, 1 mM SA, or a mock solution [sterile ddH_2_O containing an equivalent volume of dimethyl sulfoxide (DMSO)]. Treatments were applied either by foliar spray onto fully expanded leaves using solutions supplemented with 0.01% (v/v) Silwet L-77 or by root application via watering with 20 ml of solution per plant. Plants were challenge inoculated with *Pst* DC3000 24 hours after treatment.

For root bacteria–mediated systemic resistance assays, plants were root inoculated with 20 ml of bacterial suspensions (OD_600_ = 1.0) of wild-type *Pseudorhodoferax* A332, its Δ*nicAB* mutants (A332Δ*nicAB*-1 and A332Δ*nicAB*-2), or sterile water as a mock control. Challenge inoculation with *Pst* DC3000 was performed 24 hours later.

To assess resistance induced by long-term root bacterial cocultivation under sterile conditions, Col-0 seedlings were grown on one-eighth–strength Murashige and Skoog (MS) medium and either treated with sterile medium (mock) or cocultured with *Ensifer* A311 (initial OD_600_ = 0.005). After 4 weeks, leaves were challenge inoculated with *Pst* DC3000 by surface application. To evaluate disease resistance under natural soil conditions, 5-week-old Col-0 and *ugt74f2 ugt76c4* double mutant plants grown in natural soil were challenge inoculated with *Pst* DC3000.

Challenge inoculation was performed either by leaf infiltration or surface application, as specified above. For infiltration assays, *Pst* DC3000 suspensions (OD_600_ = 0.001) were infiltrated into the abaxial side of leaves 7 to 9 using a needleless 1-ml syringe between 10:00 and 11:00 a.m., and leaf samples were collected at 72 hours postinfiltration. For surface application assays, a 5-μl droplet of *Pst* DC3000 suspension [OD_600_ = 0.01, supplemented with 0.04% (v/v) Silwet L-77] was gently spread across the surface of leaves 7 to 9 using a pipette tip, and samples were collected at 4 to 5 days postinoculation.

Bacterial growth was quantified by homogenizing either three leaf disks (0.5-cm diameter) or, for surface application assays, three whole inoculated leaves (fresh weight recorded) in 1 ml of sterile Milli-Q water. Homogenates were serially diluted and plated on KB agar containing rifampicin (50 μg ml^−1^). Colony-forming units (CFU) were counted after 2 days of incubation at 28°C. Each experiment included at least 24 leaves from eight individual plants per treatment and was independently repeated at least three times with comparable results.

### Gene induction and quantification of immunity-related compounds

Sample preparation and analytical procedures were adapted from previous studies (*44*). Plants were grown under controlled short-day conditions (10-hour light/14-hour dark, 22°C, and 60% relative humidity).

For chemical treatments, 4- to 5-week-old Col-0 plants were treated with 1 mM 6-OHNA, 1 mM SA, or a mock solution, applied either by foliar spray (0.01% Silwet L-77) or by root drench (20 ml per plant). For root bacterial inoculation, an independent set of plants was treated by soil drench with 20 ml of bacterial suspensions (OD_600_ = 1.0) of wild-type *Pseudorhodoferax* A332, A332Δ*nicAB*-1, A332Δ*nicAB*-2, or sterile water.

For long-term root bacterial coculture experiments, Col-0 seedlings were grown for 4 weeks on one-eighth–strength MS medium under sterile conditions with either sterile medium (mock) or *Ensifer* A311 (initial OD_600_ = 0.005), after which root and leaf tissues were harvested for analysis. For constitutive profiling under natural soil conditions, 4- to 5-week-old Col-0 and *ugt74f2 ugt76c4* plants grown in natural soil were sampled without additional treatment.

For all treatments except long-term root bacterial coculture and constitutive profiling, systemic leaf tissues were harvested 24 hours after chemical or microbial application. For each biological replicate, the seventh, eighth, and ninth rosette leaves from 12 individual plants were pooled, immediately frozen in liquid nitrogen, and stored at −80°C. Three biological replicates were analyzed per treatment. Targeted gene expression and metabolite analyses were performed as described below. All experiments were independently repeated at least three times.

### RNA isolation and quantitative RT-PCR analysis

Total RNA was extracted from frozen leaf samples using the RNeasy Plant Mini Kit (QIAGEN). RNA quality and concentration were determined spectrophotometrically, ensuring an absorbance at 260 nm (*A*_260_)/*A*_280_ ratio greater than 1.8. Following heat denaturation at 65°C for 5 min and immediate cooling on ice, reverse transcription was performed using ReverTra Ace qPCR RT Master Mix with gDNA Remover (FSQ-301, TOYOBO). The 8 μl of reaction mixture, containing 2 μl of 4× DN Master Mix, 0.5 μg of RNA, and nuclease-free water, was incubated at 37°C for 5 min. Subsequently, 2 μl of 5× RT Master Mix was added, and the reaction was carried out at 37°C for 30 min, 50°C for 5 min, and 98°C for 5 min. The cDNA was stored at −20°C for subsequent PCR analysis.

Quantitative reverse transcription PCR (qRT-PCR) was performed using SYBR Green Mix (CW0957, Kangwei Century Biotech) on a CFX96 Real-Time PCR Detection System (Bio-Rad) (*67*). The 10-μl reaction mixture contained 5 μl of 2× SYBR Green Mix, 0.2 μl of each gene-specific primer (10 μM), 2 μl of cDNA template, and 2.6 μl of ddH2O. The thermocycling conditions were as follows: initial denaturation at 95°C for 10 min; 40 cycles of 95°C for 15 s, 60°C for 30 s, and 72°C for 30 s; followed by a melt curve analysis from 60° to 95°C. *Actin2* (*AT3G18780*) was used as the reference gene for normalization. The annealing temperature, specificity, and amplification efficiency of all primers are listed in table S3. Each sample was run in three technical replicates, and relative gene expression levels were calculated using the 2^–ΔΔ*C*t^ method (*68*), with the mock-treated group set as the calibrator. The experiment was independently repeated at least three times, with each experiment consisting of three biological replicates per treatment group.

### Structural elucidation of 6-OHNA by LC-qTOF/MS and NMR spectroscopy

The structure of the bacterial metabolite 6-OHNA was elucidated using a combination of high-resolution mass spectrometry and NMR spectroscopy. For LC–qTOF–tandem MS (MS/MS) analysis, the presence of a unique NA-derived metabolite was initially detected in culture supernatants of strains *Methylibium* A276 and *Ensifer* A311 using an Agilent 1290 ultra-performance liquid chromatography coupled to an Agilent 6550 ultrahigh-performance liquid chromatography quadrupole TOF-MS/MS (UPLC-qTOF-MS/MS), following a previously described method with minor modifications (*69*). Samples were separated on an Agilent ZORBAX Extend C18 column (1.8 μm, 2.1 mm by 100 mm; Agilent) maintained at 35°C. The mobile phase consisted of 0.1% (v/v) formic acid in water (solvent A) and 0.1% (v/v) formic acid in acetonitrile (solvent B). Separation was achieved using the following gradient at a flow rate of 0.40 ml min^−1^: 0 to 0.1 min, 1% B; 0.1 to 15.5 min, 1% to 99.5% B; 15.5 to 17.0 min, 99.5% B. The mass spectrometer was operated in positive ionization mode with the following source parameters: capillary voltage: 4000 V; gas temperature: 225°C; drying gas flow: 13 liters min^−1^; sheath gas temperature: 350°C; and sheath gas flow: 12 liters min^−1^.

For NMR analysis, 6-OHNA was purified at scale from bulk cultures of *Ensifer* A311. The compound was isolated from culture supernatants and purified to homogeneity by semipreparative high-performance liquid chromatography. The purified metabolite was dissolved in dimethyl sulfoxide–*d*_6_, and all NMR spectra were acquired on a Bruker Avance III HD 600-MHz spectrometer. ^1^H NMR and ^13^C NMR spectra were recorded at 600 and 150 MHz, respectively. Chemical shifts (δ) are reported in parts per million relative to the residual solvent signals (δ_H 2.50, δ_C 39.5), and coupling constants (*J*) are given in hertz.

### Metabolite extraction and LC-MS analysis

Metabolites were extracted from ground leaf tissues of *N. benthamiana* and *A. thaliana* using a modified protocol based on previous studies (*44*, *70*). Briefly, 100 mg of frozen leaf powder was extracted with 1 ml of 80% methanol (MeOH):water (v/v), vortexed for 5 min at room temperature, and incubated on a rotator at 4°C for 1 hour. The mixture was centrifuged at 20,000*g* for 10 min at 4°C. The supernatant was filtered through a 0.22-μm polyvinylidene fluoride filter (Millipore, Billerica, MA) and transferred to LC vials for analysis.

Quantification of 6-OHNA, SA, SAG, and Pip was conducted using an Agilent 1290 ultrahigh-performance liquid chromatograph (UPLC) system coupled with an Agilent 6495 triple quadrupole (QQQ) mass spectrometer.

For 6-OHNA, SA, and SAG quantification, samples were separated on an ACQUITY UPLC HSS C18 column (1.8 μm, 2.1 mm by 100 mm; Waters Corporation). The mobile phase consisted of deionized water with 0.1% formic acid (A) and acetonitrile with 0.1% formic acid (B). The following gradient elution program was used at a flow rate of 0.3 ml min^−1^: 0 to 0.2 min, 5% B; 0.2 to 2.5 min, 5 to 25% B; 2.5 to 4.0 min, 25 to 99% B; 4.0 to 6.0 min, 99% B; 6.0 to 6.1 min, 99 to 5% B; 6.1 to 10.0 min, 5% B. The column temperature was 35°C.

For Pip quantification, samples were separated on an ACQUITY UPLC BEH Amide column (1.7 μm, 2.1-mm inside diameter by 100 mm; Waters Corporation). The mobile phases consisted of water with 10 mM ammonium formate and 0.125% formic acid (A) and acetonitrile with 10 mM ammonium formate and 0.125% formic acid (B). A gradient elution was performed at a flow rate of 0.4 ml min^−1^, as follows: 0 to 1.5 min, 100% B; 1.5 to 6.0 min, 100 to 40% B; 6.0 to 8.0 min, 40% B; 8.0 to 8.5 min, 40 to 30% B; 8.5 to 9.5 min, 30 to 100% B; 9.5 to 12 min, 100% B. The column temperature was also 35°C.

All metabolites were quantified in multiple reaction monitoring (MRM) mode. 6-OHNA and Pip were analyzed in positive ionization mode, whereas SA and SAG were analyzed in negative ionization mode. Nitrogen (N_2_) was used as both drying gas (12 liters min^−1^ at 200°C) and sheath gas (12 liters min^−1^ at 350°C). The nebulizer pressure was set to 30 psi (206.8 kPa), and the fragmentor voltage was 380 V. Optimized metabolite-specific MRM transitions and collision energies are listed in table S4. Data were processed using MassHunter software B07.01 (Agilent Technologies).

### Root exudate collection and LC-MS analysis

Root exudates from *A. thaliana* genotypes Col-0, *nic1-1*, *ugt74f2*, and *ugt74f2 ugt76c4* were collected at two developmental stages (14- and 21-day-old seedlings). Plants were grown under short-day conditions (10-hour light/14-hour dark, 22°C, and 50% relative humidity) in a controlled greenhouse. At each time point, plants were gently transferred to sterile Milli-Q water and incubated for 24 hours to allow the release of root exudates. The exudates were collected and concentrated by lyophilization. The dried samples were reconstituted in 100 μl of 50% acetonitrile, vortexed thoroughly, and extracted at 4°C for 1 hour. After centrifugation at 20,000*g* for 5 min, the supernatant was subjected to LC-MS analysis.

Quantification of NA-related metabolites was performed using an Agilent 1290 UPLC system coupled to an Agilent 6495 QQQ mass spectrometer, following a previously described method with minor modifications (*71*). Separation was achieved on an ACQUITY UPLC BEH Amide column (1.7 μm, 2.1 mm by 100 mm) maintained at 35°C. Both mobile phases contained 0.15% ammonium hydroxide and 5 mM ammonium acetate; solvent A consisted of 50% acetonitrile in water, and solvent B consisted of 95% acetonitrile in water. A linear gradient was applied at 0.35 ml min^−1^ as follows: 0 to 0.5 min, 98% B; 0.5 to 10.0 min, 98 to 75% B; 10.0 to 11.0 min, 75% B; 11.0 to 11.01 min, 75 to 98% B; 11.01 to 16.0 min, 98% B.

The mass spectrometer was operated in MRM mode using nitrogen as the drying gas (12 liters min^−1^ at 200°C) and sheath gas (12 liters min^−1^ at 350°C), with a nebulizer pressure of 30 psi (206.8 kPa) and a fragmentor voltage of 380 V. Optimized MRM transitions and collision energies are provided in table S4. Data analysis was performed using MassHunter software B07.01 (Agilent Technologies).

### Radiotracer biosynthesis and transport assay

[^14^C]-labeled 6-OHNA was biosynthesized by incubating strains *Methylibium* A276 and *Ensifer* A311 with [^14^C]-NA or [^14^C]-NAM for 8 hours. The resulting [^14^C]–6-OHNA–containing supernatant was then applied to the roots of 14-day-old *Arabidopsis* Col-0 plants. After 24 hours of cocultivation, plants were harvested for analysis.

The distribution of radiolabeled compounds was determined by analyzing three samples: the prefeeding bacterial culture supernatant, the postfeeding supernatant after cocultivation with plant roots, and leaf tissues of Col-0 plants. Extraction and TLC analysis of plant samples were performed according to a previous method (*10*) with minor modifications. A total of 15-μl aliquots of supernatant samples or leaf extracts was spotted onto POLYGRAM TLC plates (Macherey-Nagel). Leaf extracts were prepared by homogenizing disks in 250 μl of 20 mM sodium diethyldithiocarbamate followed by centrifugation (5 min at 20,000*g*).

Chromatography was performed using a mobile phase of *n*-butanol:acetic acid:water [2:1:1 (v/v/v)]. Radioactive metabolites were visualized and quantified using an InstantImager radio scanner (Packard Instrument Company Inc.).

### In vitro antibacterial activity assay

*Pst* DC3000 was precultured overnight in KB liquid medium supplemented with rifampicin (50 μg ml^−1^) at 28°C with shaking at 220 rpm. The culture was diluted to an OD_600_ of 0.001 (∼10^6^ CFU ml^−1^) in fresh KB medium, and 200 μl aliquots were transferred into a 96-well plate. Test compounds (6-OHNA and SA) were prepared as 100 mM stock solutions in DMSO and added to bacterial cultures at final concentrations of 10, 100, and 1,000 μM. A solvent control (DMSO matched to each treatment) and an untreated control (medium only) were included. SA served as a positive control. Plates were incubated at 28°C with continuous shaking (220 rpm), and bacterial growth was monitored by measuring OD_600_ at 0, 12, 18, 24, and 36 hours postinoculation using a microplate reader.

### Gene knockout in root bacteria via conjugation-mediated plasmid delivery

Gene knockouts in root bacteria were generated through conjugation-mediated delivery of a suicide plasmid, followed by homologous recombination and counterselection. Briefly, a 1.5-kb DNA fragment flanking the target gene was PCR amplified using primer pairs listed in table S3 and cloned into a modified pK18mobsacB suicide vector conferring chloramphenicol resistance. The resulting plasmid was electroporated into *E. coli* S17-1 *pir* to create the donor strain.

For conjugation, donor and recipient strains were mixed at a 1:1 ratio and coincubated on nonselective LB agar for 16 to 18 hours at 28°C. The cell mixture was then plated onto LB agar supplemented with chloramphenicol (50 μg ml^−1^) to select for transconjugants. To isolate clones that had excised the plasmid via a second homologous recombination event, transconjugants were streaked onto LB agar containing 5% (w/v) sucrose. This sucrose counterselection was repeated for two to three rounds. Successful gene deletion was confirmed by PCR analysis of the target locus using verification primers provided in table S3 and verified by Southern blot analysis.

### Root colonization assay

Root colonization assays were performed using a previously described protocol with minor modifications (*72*). *A. thaliana* Col-0 seeds were surface sterilized and stratified at 4°C for 2 days and then grown on ½ MS medium under short-day conditions (10-hour light/14-hour dark, 22°C, and 50% relative humidity). Twelve-day-old seedlings of Col-0 were inoculated with either wild-type *Pseudorhodoferax* A332 or the isogenic Δ*nicAB-1* mutant (OD_600_ = 1.0). Seedling-bacteria cocultivation was maintained under the same short-day growth condition. Root-associated bacterial colonization was quantified at 4 days postinoculation for Col-0.

For CFU quantification, whole plants were collected and washed three times with 1× PBS buffer (pH 7.4) at 180 rpm to remove weakly attached bacteria. Washed roots were homogenized in sterile ddH_2_O. The resulting homogenates were serially diluted and plated onto ½ TSB agar plates containing ampicillin (100 μg ml^−1^). Plates were incubated at 28°C for 2 days. Colony numbers were counted, and bacterial colonization levels were normalized to root fresh weight and presented as log(CFU/mg).
