# Supplementary material for: A vitamin B3–driven root bacterial metabolite primes systemic immunity in Arabidopsis
Source: Sci Adv. 2026 Aug 26;12(35):eaef8959. doi: 10.1126/sciadv.aef8959 (PMC13510662; doi:10.1126/sciadv.aef8959)
Supplement: Supplementary file 1 [file sciadv.aef8959_sm.pdf]

Science Advances
AAAS

Supplementary Materials for

A vitamin B_3–driven root bacterial metabolite primes systemic immunity in *Arabidopsis*

Xuemei Wang *et al.*

Corresponding author: Yang Bai, ybai@pku.edu.cn; Guodong Wang, gdwang@genetics.ac.cn

Sci. Adv. **12**, eaef8959 (2026)
DOI: 10.1126/sciadv.aef8959

The PDF file includes:

Figs. S1 to S18
Legends for tables S1 to S4

Other Supplementary Material for this manuscript includes the following:

Tables S1 to S4

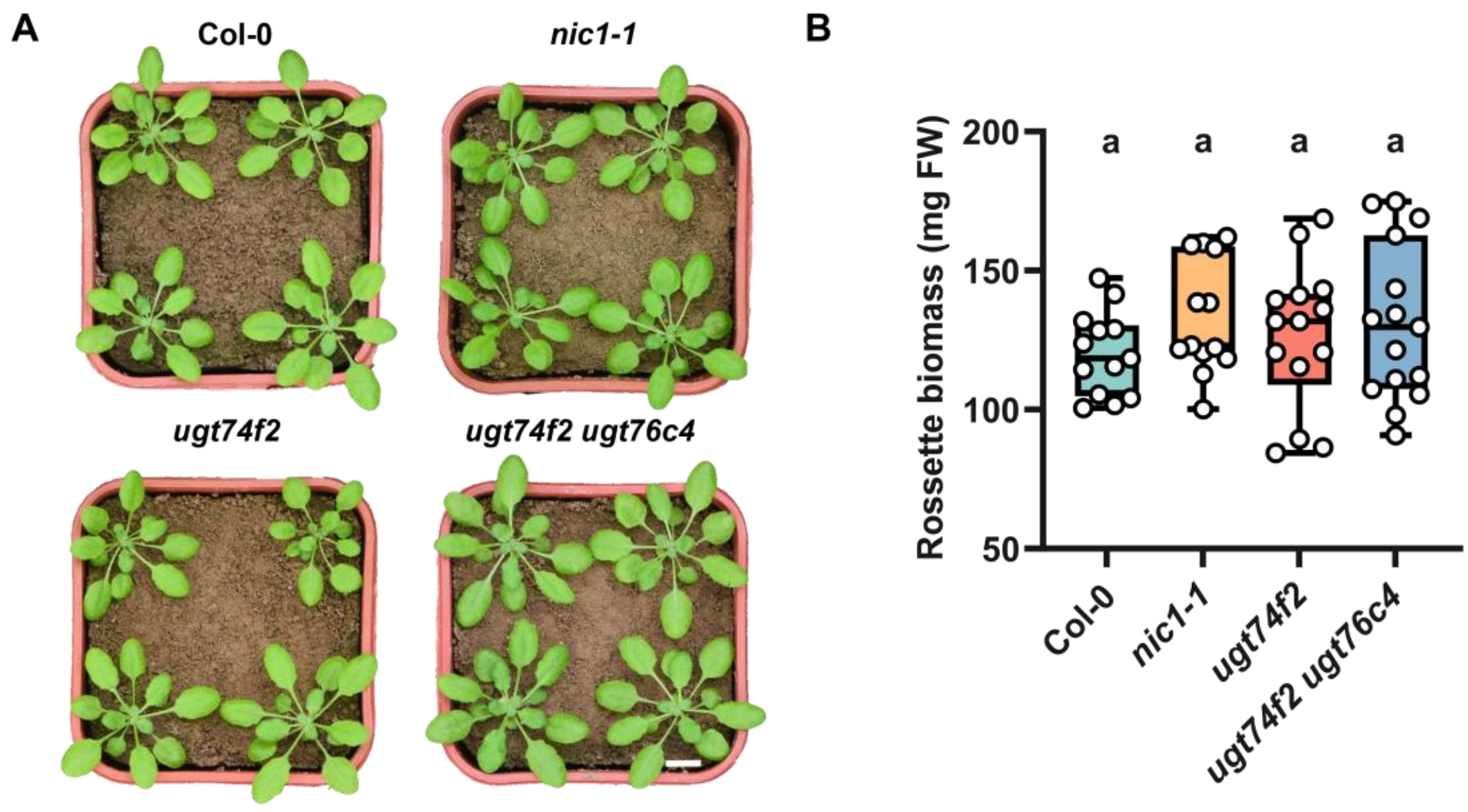

Fig. S1. Normal growth of VB_3-related mutants under natural soil conditions.

(A) Representative images of Col-0, *nic1-1*, *ugt74f2*, and *ugt74f2 ugt76c4* plants grown for six weeks in natural soil under short-day conditions. Scale bar, 1 cm.

(B) Aboveground fresh weight (FW) of the indicated genotypes. Data are shown as box plots with individual data points (Col-0, $n = 13$; *nic1-1*, $n = 13$; *ugt74f2*, $n = 14$; *ugt74f2 ugt76c4*, $n = 15$). No significant differences were detected (one-way analysis of variance (ANOVA) followed by Tukey's multiple-comparison test; $p > 0.05$).

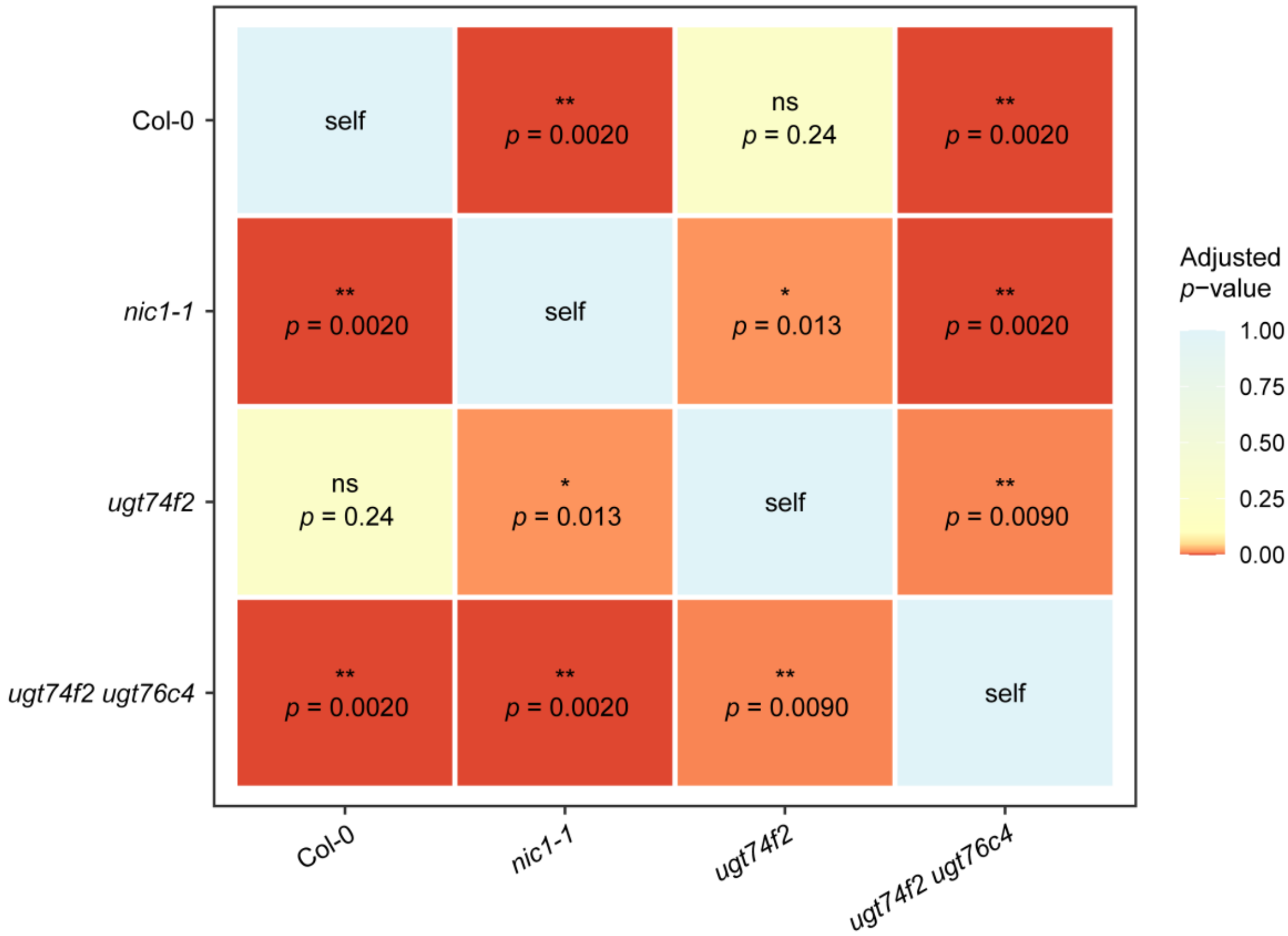

Fig. S2. Pairwise community dissimilarity comparison across *Arabidopsis* genotypes via PERMANOVA.

Pairwise permutational multivariate analysis of variance (PERMANOVA) based on Bray-Curtis dissimilarity, showing BH-adjusted *p*-values for root microbiota compositional differences between *Arabidopsis thaliana* genotypes Col-0, *nic1-1*, *ugt74f2*, and *ugt74f2 ugt76c4*.The diagonal represents self-comparison within each genotype. Color gradient corresponds to the magnitude of adjusted *p*-values, with darker red indicating higher statistical significance; ns, not significant; *, adjusted $p < 0.05$; **, adjusted $p < 0.01$. All *p*-values were corrected for multiple comparisons using the Benjamini-Hochberg (BH) method.

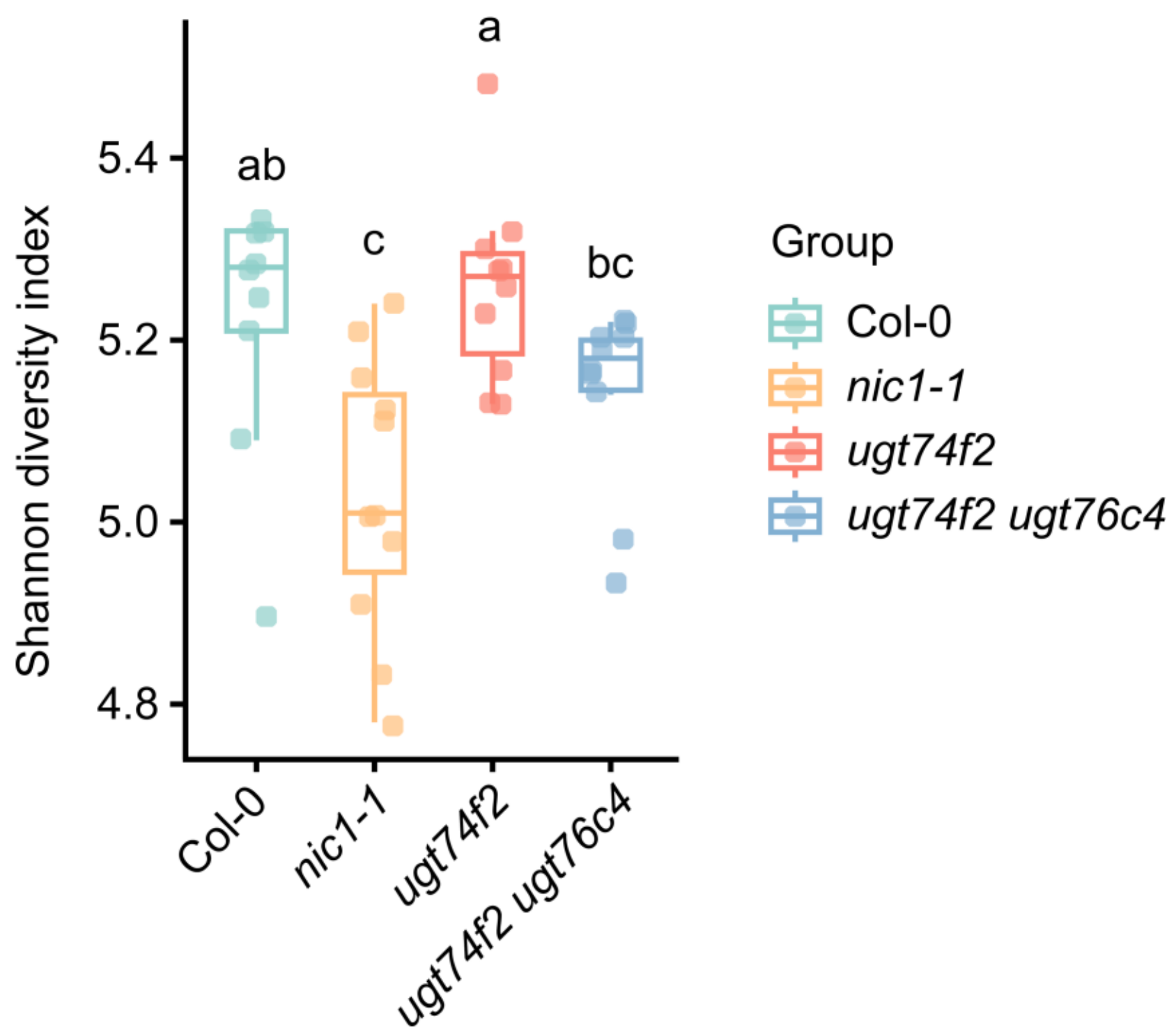

Fig. S3. Root microbiota α-diversity across genotypes.

Shannon diversity indices of root-associated microbiota calculated from 16S rRNA gene sequencing. Each dot represents an individual sample; boxes indicate the interquartile range with median values, and whiskers extend to 1.5× the interquartile range. Statistical significance was assessed by one-way ANOVA. Sample sizes: Col-0 (*n* = 9), *nic1-1* (*n* = 11), *ugt74f2* (*n* = 10), and *ugt74f2 ugt76c4* (*n* = 10).

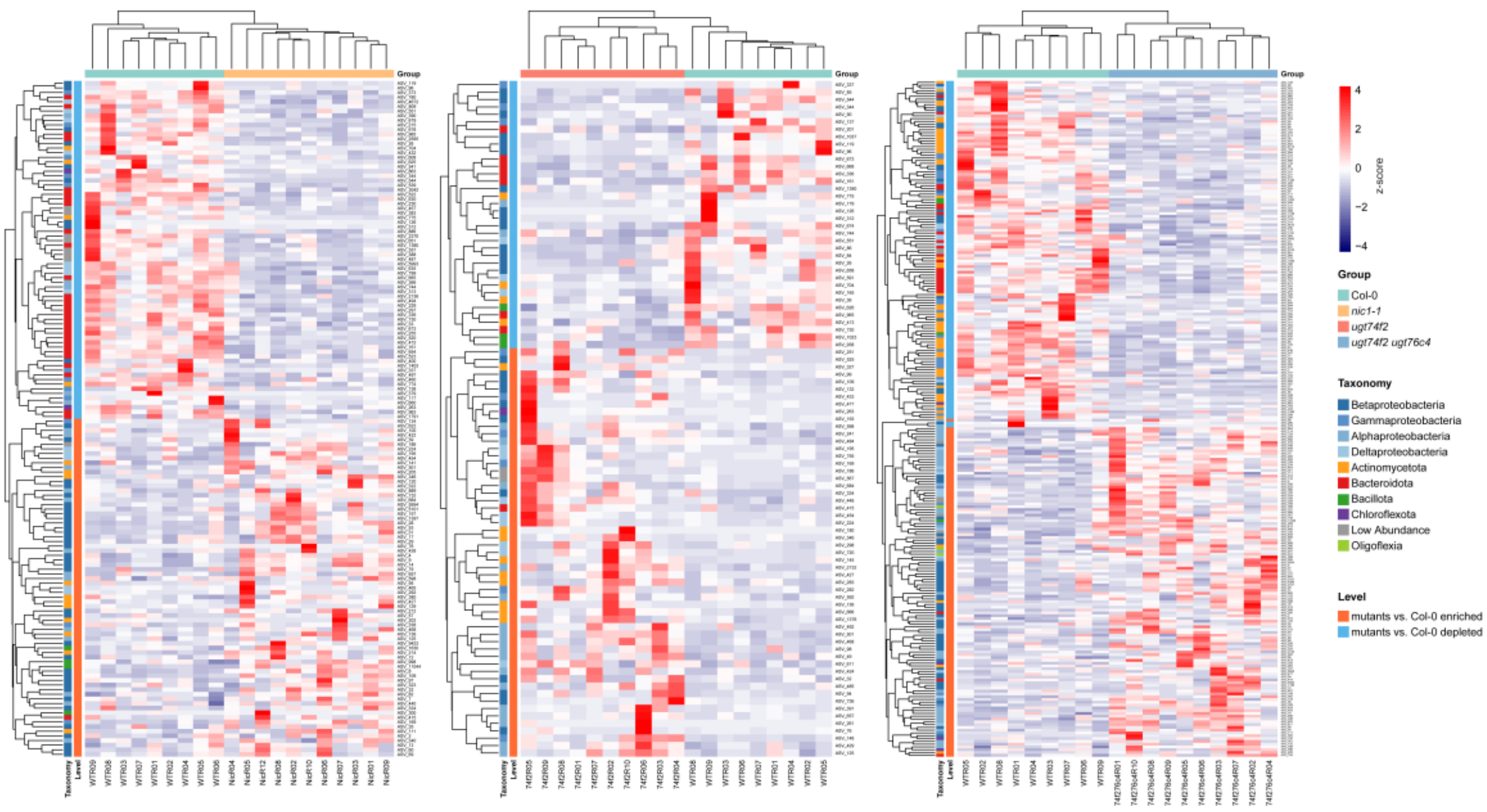

Fig. S4. Altered root microbiota composition in NA metabolism mutants.

Heatmap of differentially abundant amplicon sequence variants (ASVs) between Col-0 and mutants defective in NA metabolism. The three panels from left to right represent comparisons of *nic1-1* versus Col-0, *ugt74f2* versus Col-0, and *ugt74f2 ugt76c4* versus Col-0, respectively. Columns represent individual plant samples grouped by genotype; rows correspond to distinct ASVs with hierarchical clustering shown on the left. ASV abundances are *z*-score normalized; red and blue indicate enrichment and depletion, respectively, in mutants relative to Col-0. Row side colors denote ASV taxonomic classification, while the outer annotation marks ASVs significantly enriched (orange) or depleted (cyan) in mutants compared to Col-0. ASVs are annotated and color-coded by phylum as indicated in the legend. Sample sizes: Col-0 (n = 9), *nic1-1* (n = 11), *ugt74f2* (n = 10), and *ugt74f2 ugt76c4* (n = 10).

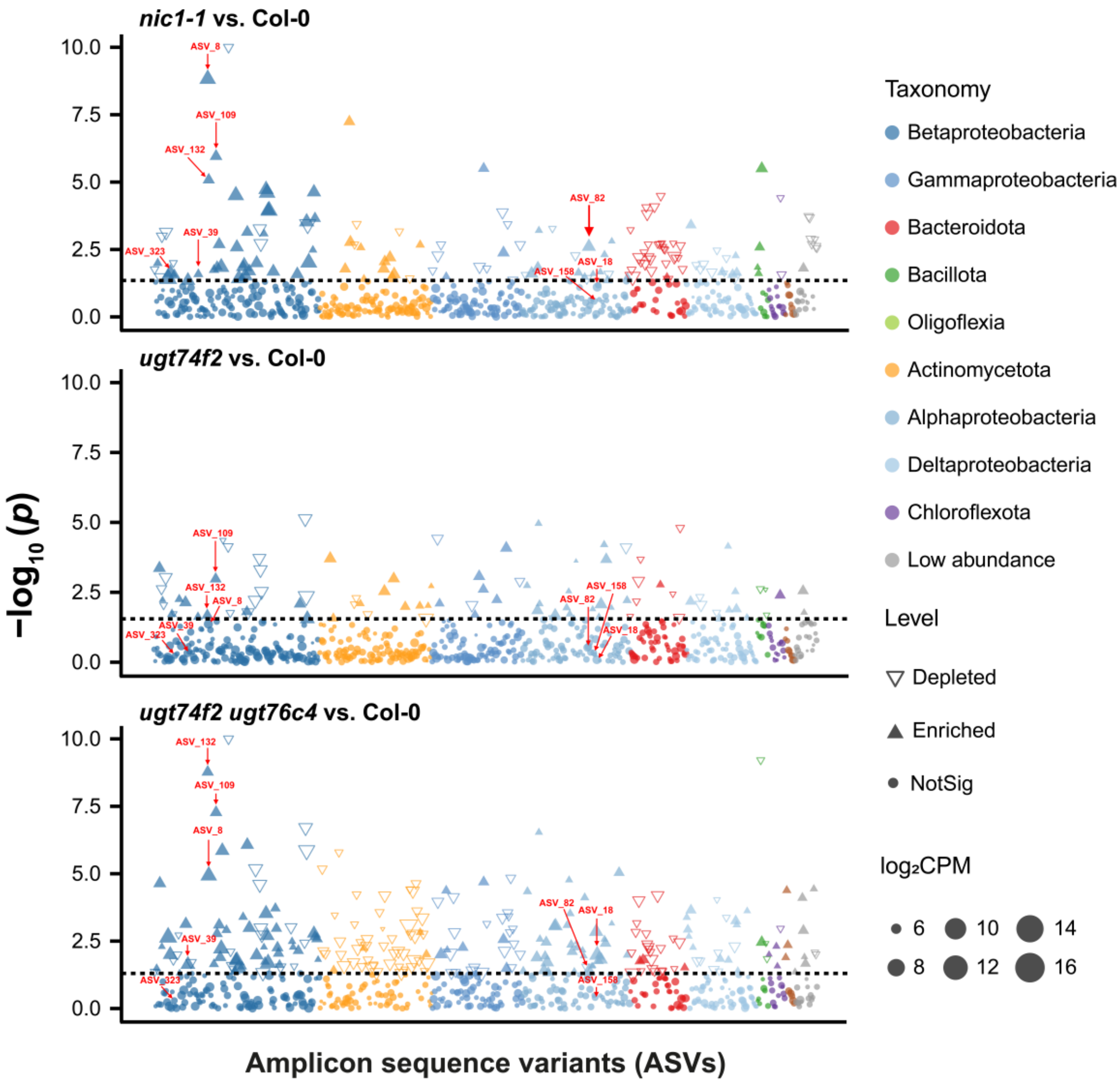

Fig. S5. Taxon-specific modulation of root-associated ASVs in NA metabolism mutants.

Manhattan plot showing ASVs changes within the root microbiota of NA metabolism mutants. Each dot or triangle represents an individual ASV; filled and open triangles denote significantly enriched or depleted ASVs, respectively ($p < 0.05$, false discovery rate (FDR) < 0.2). ASVs are grouped by taxonomic affiliation and color-coded at the phylum or class level. Representative ASVs corresponding to isolates harboring the *nic* biosynthetic gene cluster are highlighted and labeled with red arrows. Abundance is shown as counts per million (CPM).

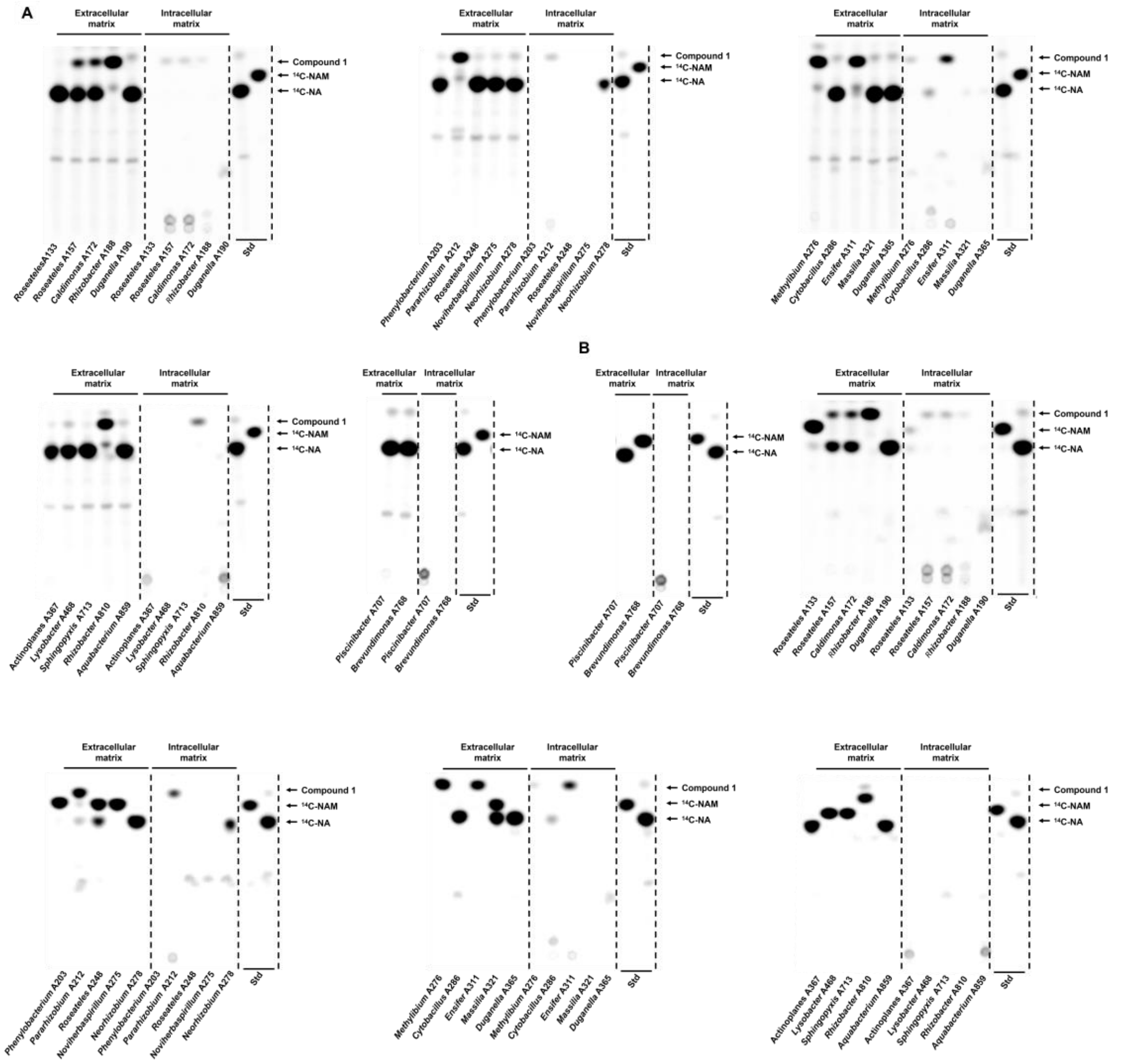

Fig. S6. Root bacterial conversion of VB_3 precursors revealed by radio-TLC. Additional VB_3-associated bacterial strains were cultured and analyzed as described in Fig. 3A. Radiolabeled metabolites were separated by TLC and visualized by phosphor imaging. The migration positions of [^{14}C]-NA and [^{14}C]-NAM reference standards (Std) are marked at the right, alongside an uncharacterized metabolite (Compound 1).

(A) Radio-TLC analysis of selected strains after incubation with [^{14}C]-NA.

(B) Radio-TLC analysis of selected strains after incubation with [^{14}C]-NAM.

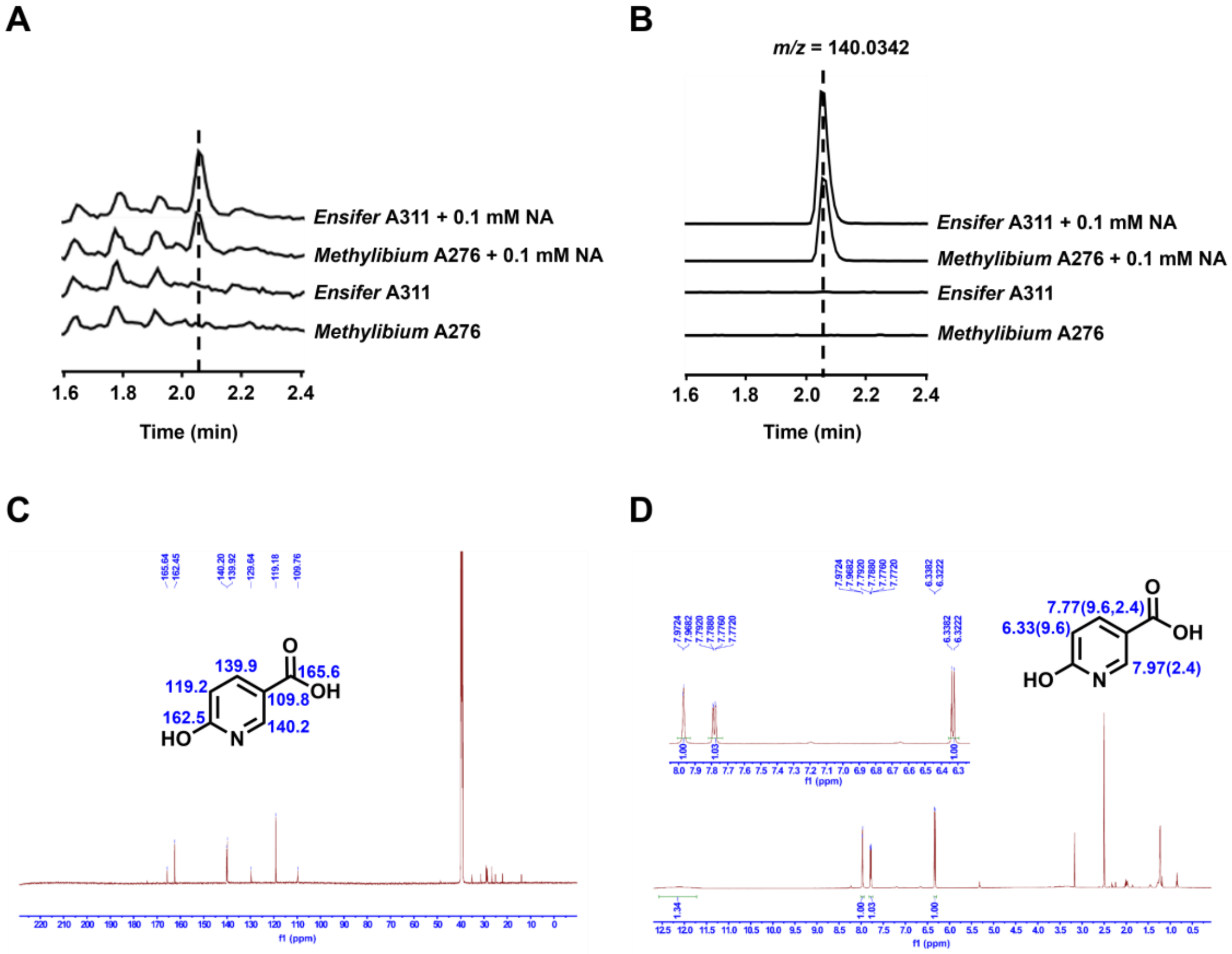

Fig. S7. Structural characterization of NA-derived 6-OHNA.

(A) Total ion chromatograms (TICs) of culture supernatants from *Methylibium* A276 and *Ensifer* A311 grown with or without NA, revealing an NA-derived differential peak.

(B) Extracted ion chromatograms (EICs) at *m/z* 140.0342 confirming the presence of the NA-derived metabolite exclusively in NA-supplemented cultures.

(C, D) ^{13}C NMR spectrum **(C)** and ^{1}H NMR spectrum **(D)** of the NA-derived metabolite purified from *Ensifer* A311. Atom numbering corresponds to NMR assignments in Table S2A.

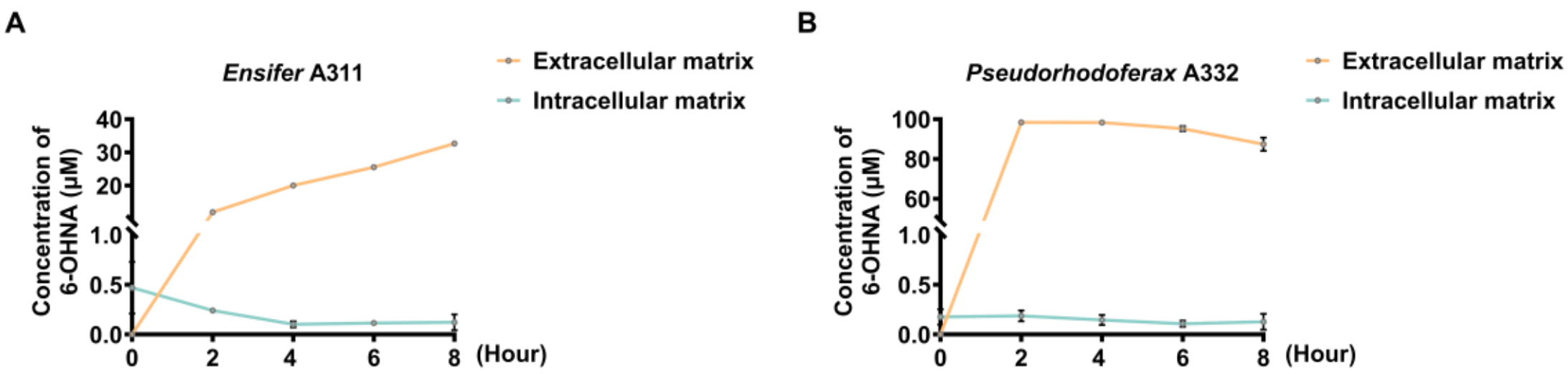

Fig. S8. Time-course quantification of 6-OHNA production in two *nic*-containing strains.

1 mL of bacterial culture (OD_{600} = 1) was supplemented with 100 μM NA, and the concentration of 6-OHNA was quantified in extracellular (supernatant) and intracellular (cell pellet) fractions at the indicated time points via targeted LC-QQQ-MS. Assays were performed for *Ensifer* A311 **(A)** and *Pseudorhodoferax* A332 **(B)**. Data are presented as mean ± s.d. (*n* = 3).

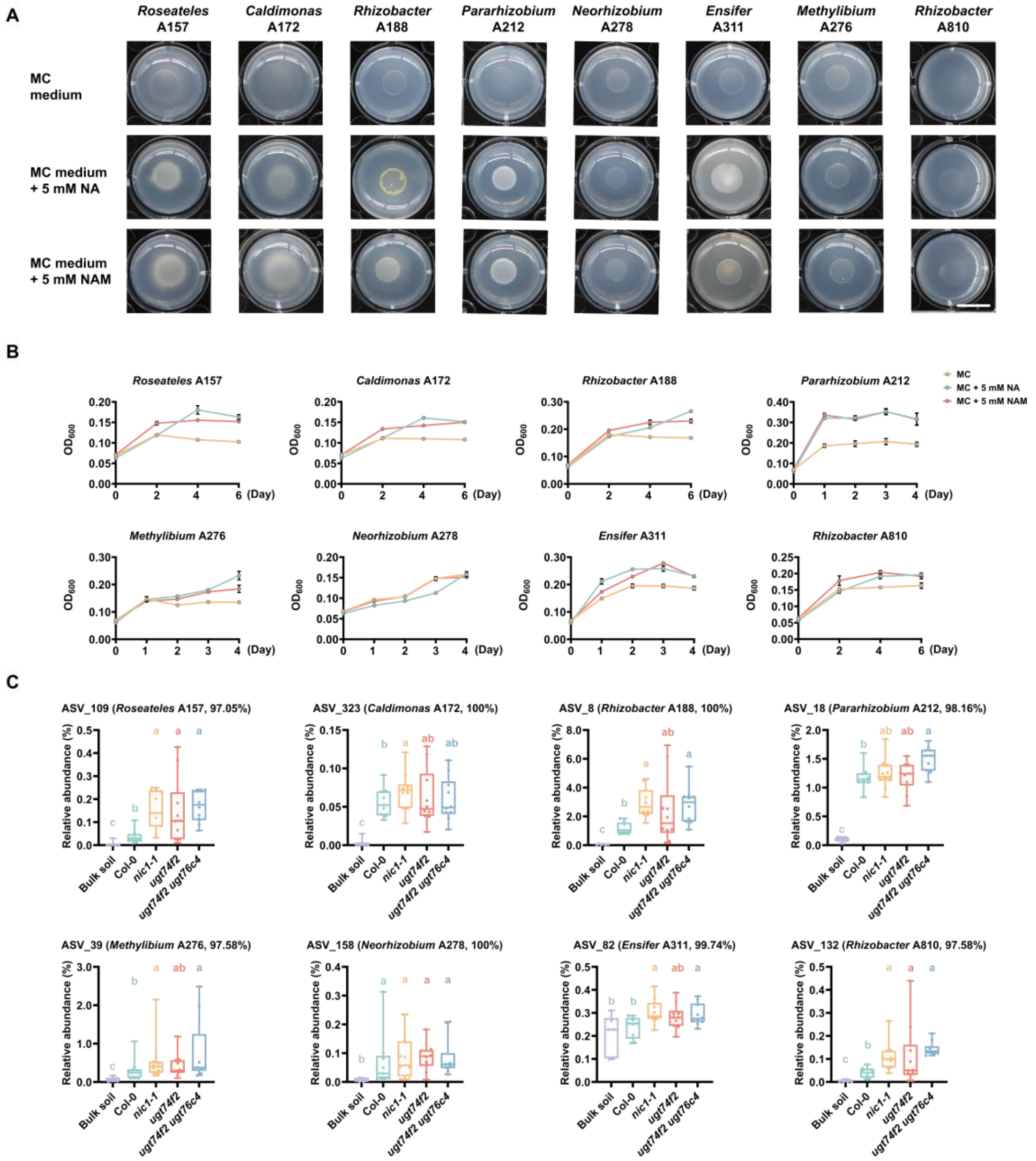

Fig. S9. Growth characteristics and root enrichment of VB$_3$-associated *nicAB-carrying* strains.

(A) Growth of eight *nicAB*-encoding strains on MC minimal medium, with or without supplementation of 5 mM NA or 5 mM NAM. Representative images are shown. Scale bar, 1 cm.

(B) Growth curves of the same strains in liquid MC medium. Optical density at 600 nm (OD_{600}) was measured over time for slow- and fast-growing strains as indicated. Data are mean ± s.d. (n = 4).

(C) Relative abundance of *nicAB*-carrying strains in bulk soil and in roots of

Arabidopsis thaliana genotypes Col-0, *nic1-1*, *ugt74f2*, and *ugt74f2 ugt76c4*. Sample sizes are indicated below. Statistical significance was assessed by pairwise edgeR comparisons (FDR < 0.2; $|\log_2$ fold change$| > \log_2 1.2$); different letters denote significant differences.

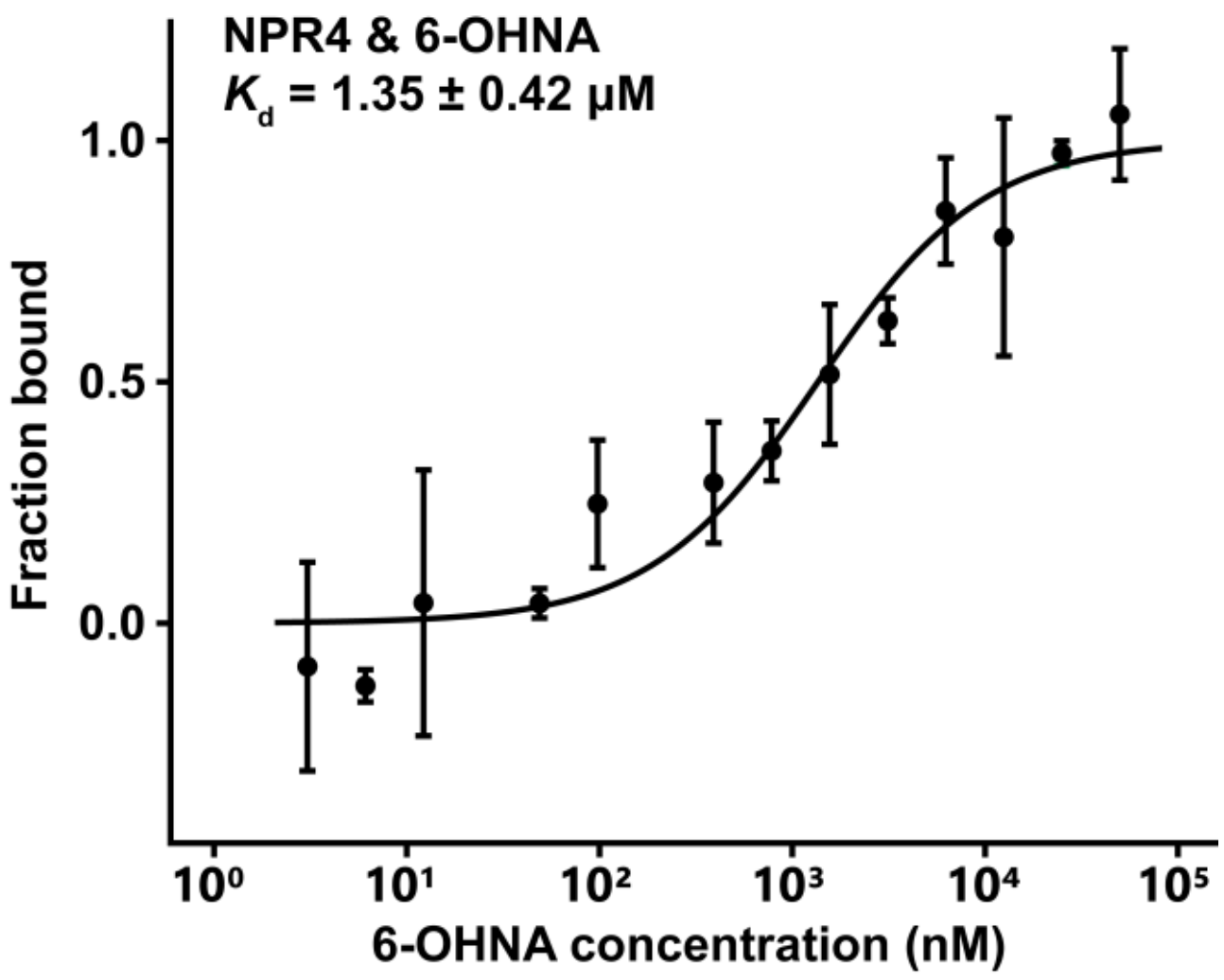

Fig. S10. 6-OHNA interacts with NPR4 protein *in vitro* as detected by MST assay. Microscale thermophoresis (MST) was used to quantify the binding affinity in vitro between 6-OHNA and NPR4. The fitted dissociation constant K_d = 1.35 ± 0.42 μM. Data are mean ± s.d. of three independent replicates (n = 3).

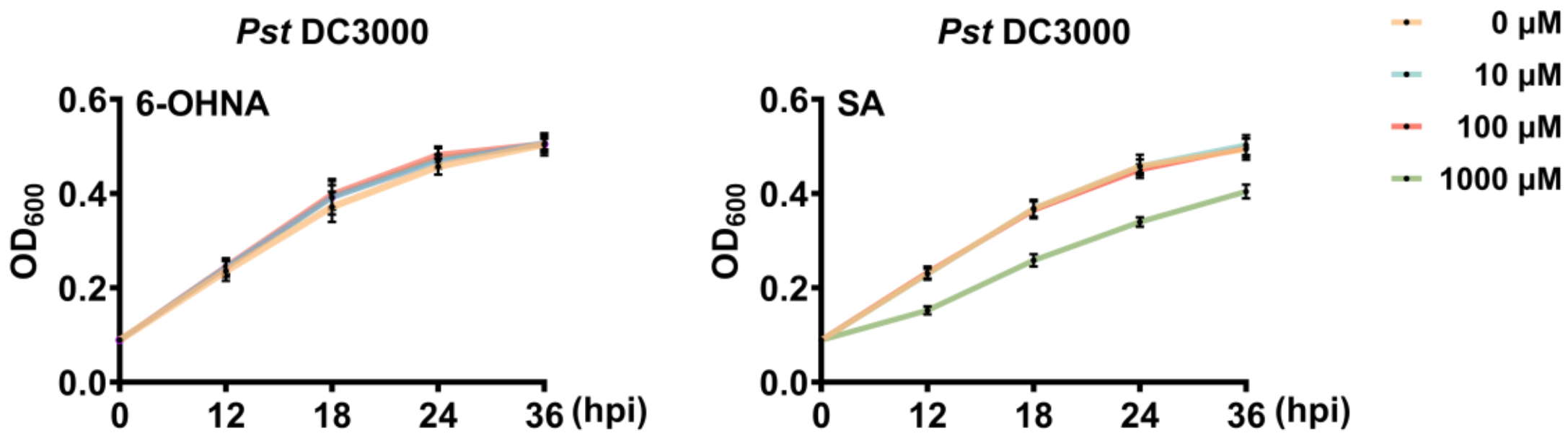

Fig. S11. 6-OHNA does not inhibit pathogen growth *in vitro*.

Growth of *Pst* DC3000 in the presence of 6-OHNA or SA, measured as OD_{600} over time. Data are mean ± s.d. of six biological replicates and are representative of three independent experiments. Hpi, hours post inoculation.

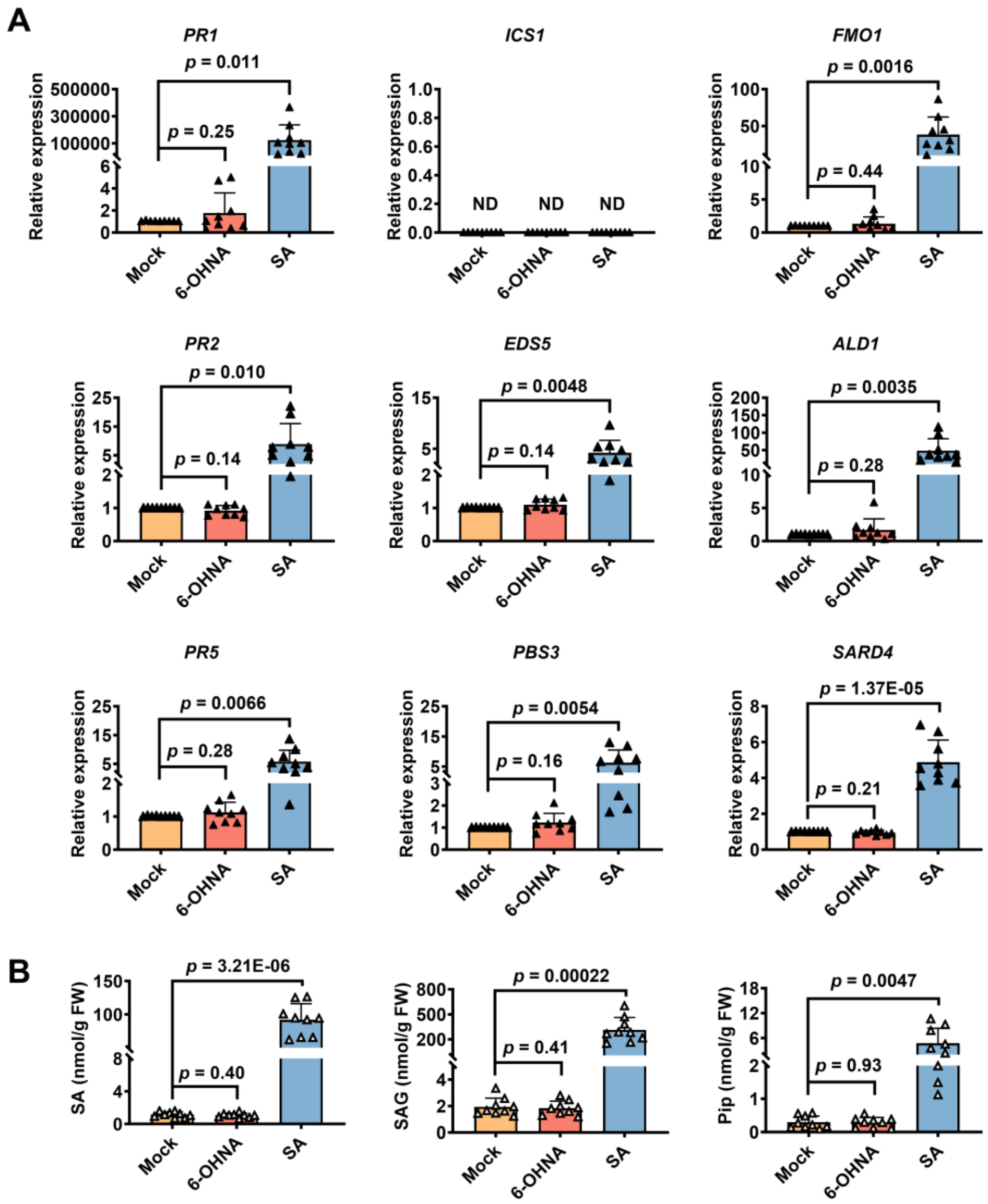

Fig. S12. 6-OHNA fails to activate defense-associated gene expression and metabolite accumulation in the *sid2-2* mutant.

(A) RT-qPCR analysis of relative transcript levels for immunity-related genes (*PR1*, *PR2*, *PR5*, *ICS1*, *EDS5*, *PBS3*, *FMO1*, *ALD1*, and *SARD4*) in *sid2-2* leaves treated with 1 mM 6-OHNA, 1 mM SA or mock. ND, not detected.

(B) The content of defense-associated metabolites (SA, SAG, and Pip) in *sid2-2* leaf tissues following mock, 1 mM 6-OHNA, or 1 mM SA treatment. FW, fresh weight.

Data represent mean ± s.d. (n = 9 from three independent experiments). Statistical significance was assessed using two-tailed paired Student's t-tests, with exact p-values indicated.

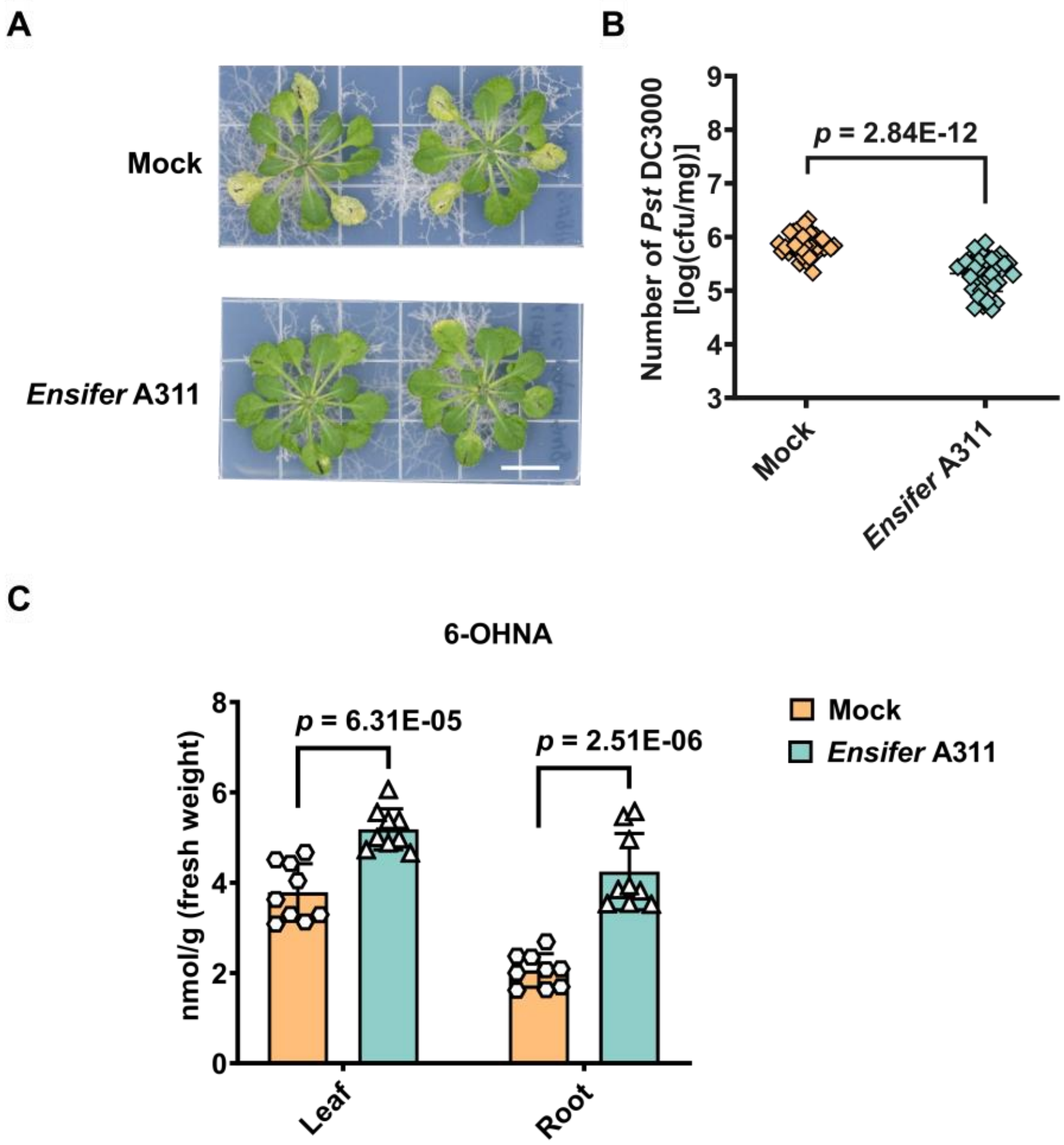

Fig. S13. Root bacterial colonization enhances disease resistance and 6-OHNA accumulation.

(A) Disease symptoms in Col-0 leaves at 5 dpi with *Pst* DC3000 following 4-week co-cultivation with *Ensifer* A311 or mock under gnotobiotic conditions. Representative images from three independent experiments are shown. Scale bar, 1.5 cm.

(B) Bacterial growth of *Pst* DC3000 in Col-0 leaves pretreated with *Ensifer* A311 or mock. Data are pooled from three independent experiments and shown as mean ± s.d. (n = 36 per group). Statistical significance was assessed using unpaired two-tailed Student's *t*-tests.

(C) Accumulation of 6-OHNA in roots and leaves following co-cultivation with *Ensifer* A311 or mock. Data are mean ± s.d. (n = 9). Statistical significance was evaluated using paired two-tailed Student's *t*-tests. FW, fresh weight.

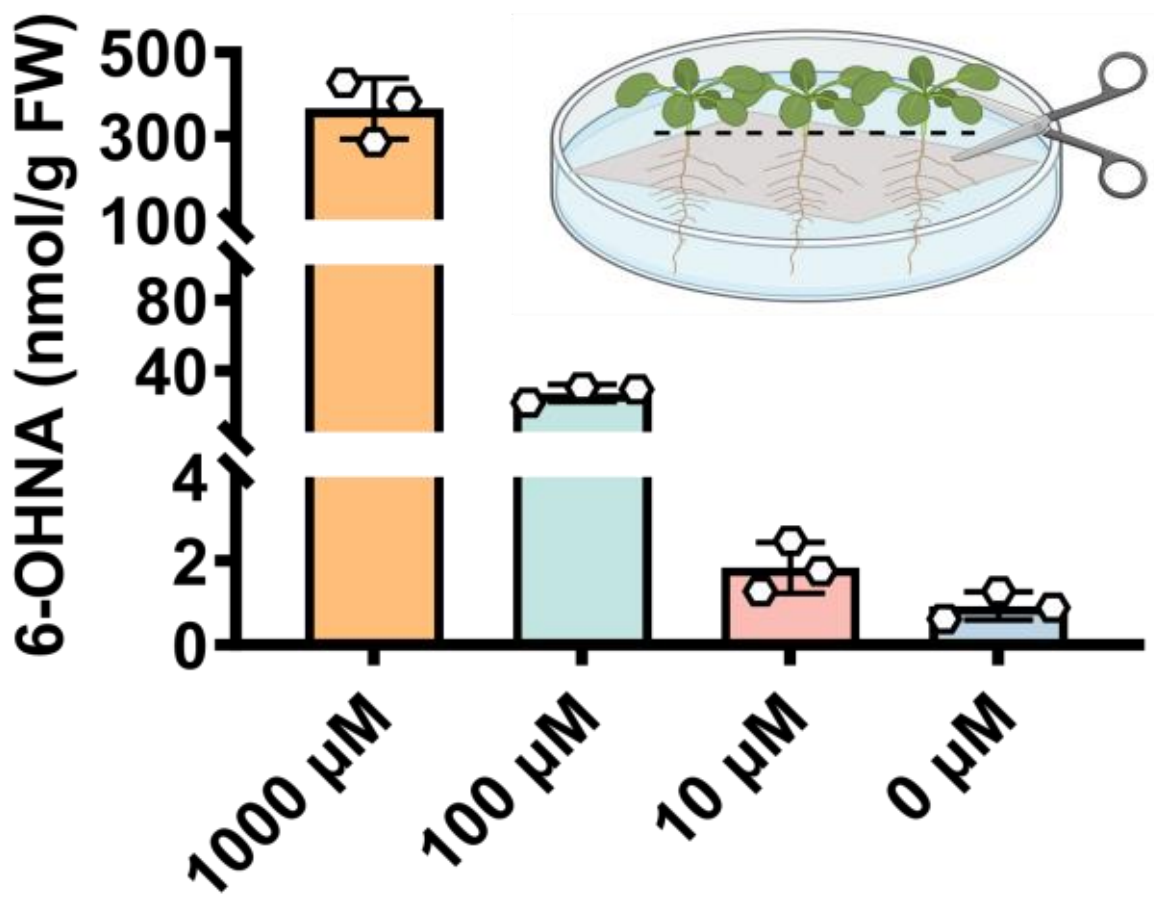

Fig. S14. Root-to-shoot transport of 6-OHNA.

Schematic illustration of the co-cultivation system used to assess long-distance transport. Total 6-OHNA levels in leaves were quantified by LC-QQQ-MS 2 days after root treatment. Data are presented as mean ± s.d. of three biological replicates. FW, fresh weight. Created in BioRender. Wang, X. (2026) https://BioRender.com/jjhg2u5.

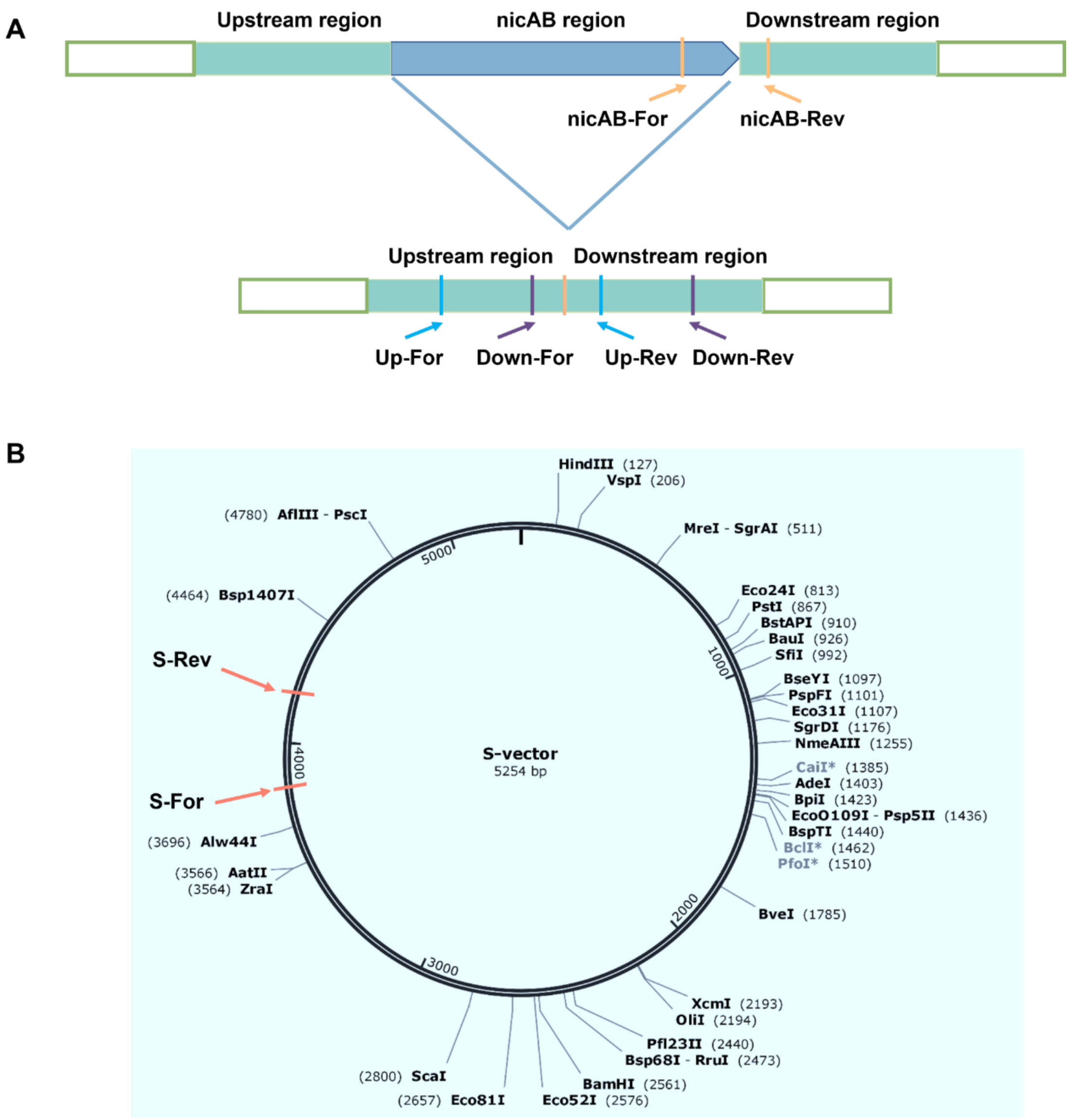

Fig. S15. Allelic exchange strategy for *nicAB* deletion in *Pseudorhodoferax* A332.

(A) Schematic of the *nicAB* knockout strategy. Upstream and downstream homologous regions flanking the *nicAB* locus were cloned into a suicide plasmid to enable allelic exchange by homologous recombination. Primer positions used for genotyping are indicated and allow discrimination between wild-type and deletion alleles.

(B) Map of the suicide plasmid used for recombination. The plasmid contains upstream and downstream homologous arms and lacks a functional origin of replication in *Pseudorhodoferax* A332. Primer pair S-F/S-R, located within the plasmid backbone, was used to verify plasmid excision following double-crossover recombination. All primer pairs shown were used for PCR validation assays described in Table S3.

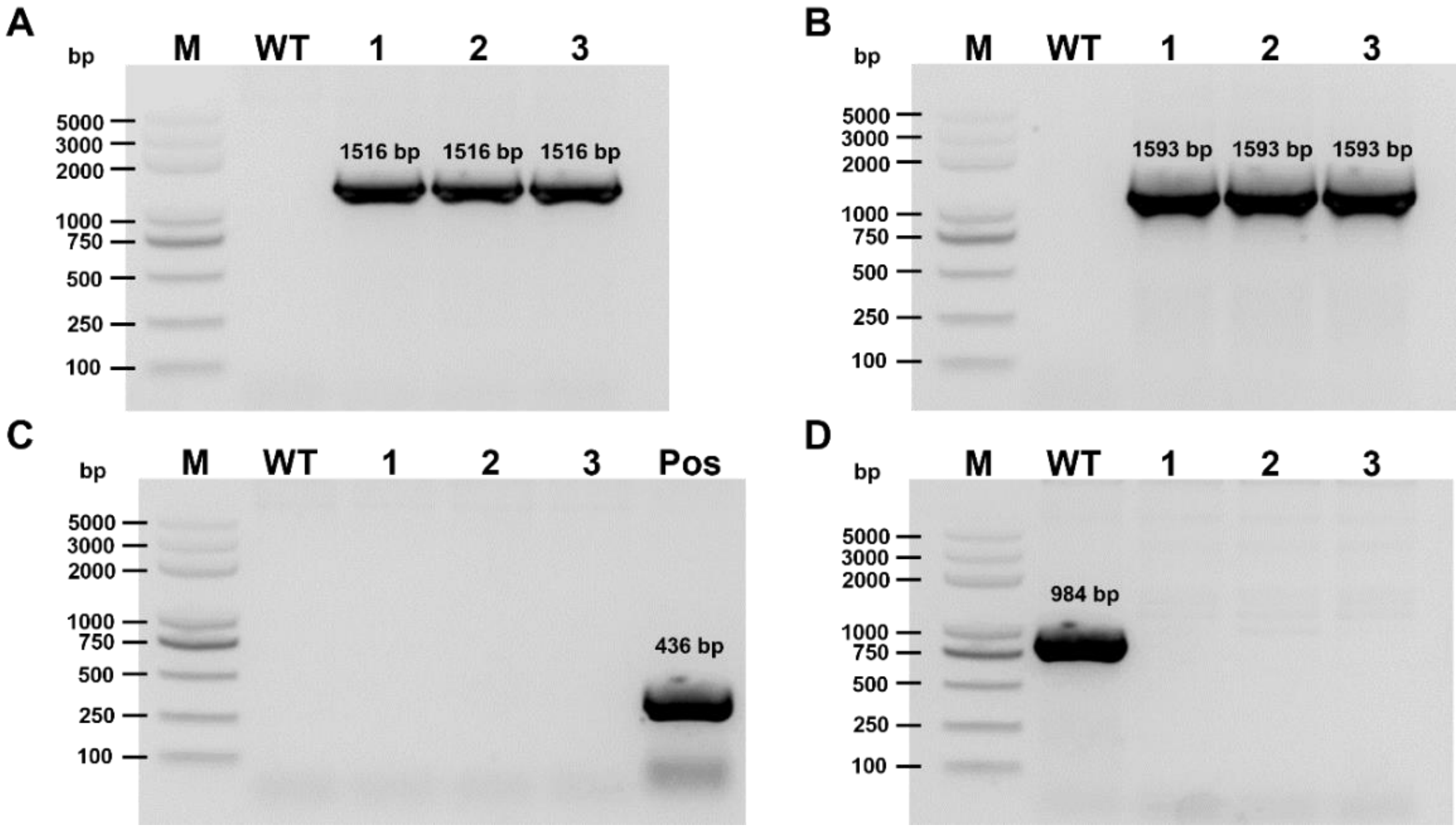

Fig. S16. PCR confirmation of *nicAB* deletion in *Pseudorhodoferax* A332.

(A, B) PCR analysis using primer pairs flanking the *nicAB* locus (shown in Fig. S14) produced the expected deletion-specific fragments in *Pseudorhodoferax* A332Δ*nicAB* mutants (-1, -2 and -3), consistent with double-crossover recombination at both homology arms.

(C) PCR analysis using plasmid-specific primers (S-F/S-R) detected an amplification product only in the plasmid control, confirming complete excision of the suicide vector in the mutants.

(D) PCR using primers spanning the *nicAB* coding region (nicAB-F/nicAB-R) amplified a fragment exclusively in the wild-type strain, verifying deletion of *nicAB* in the mutants.

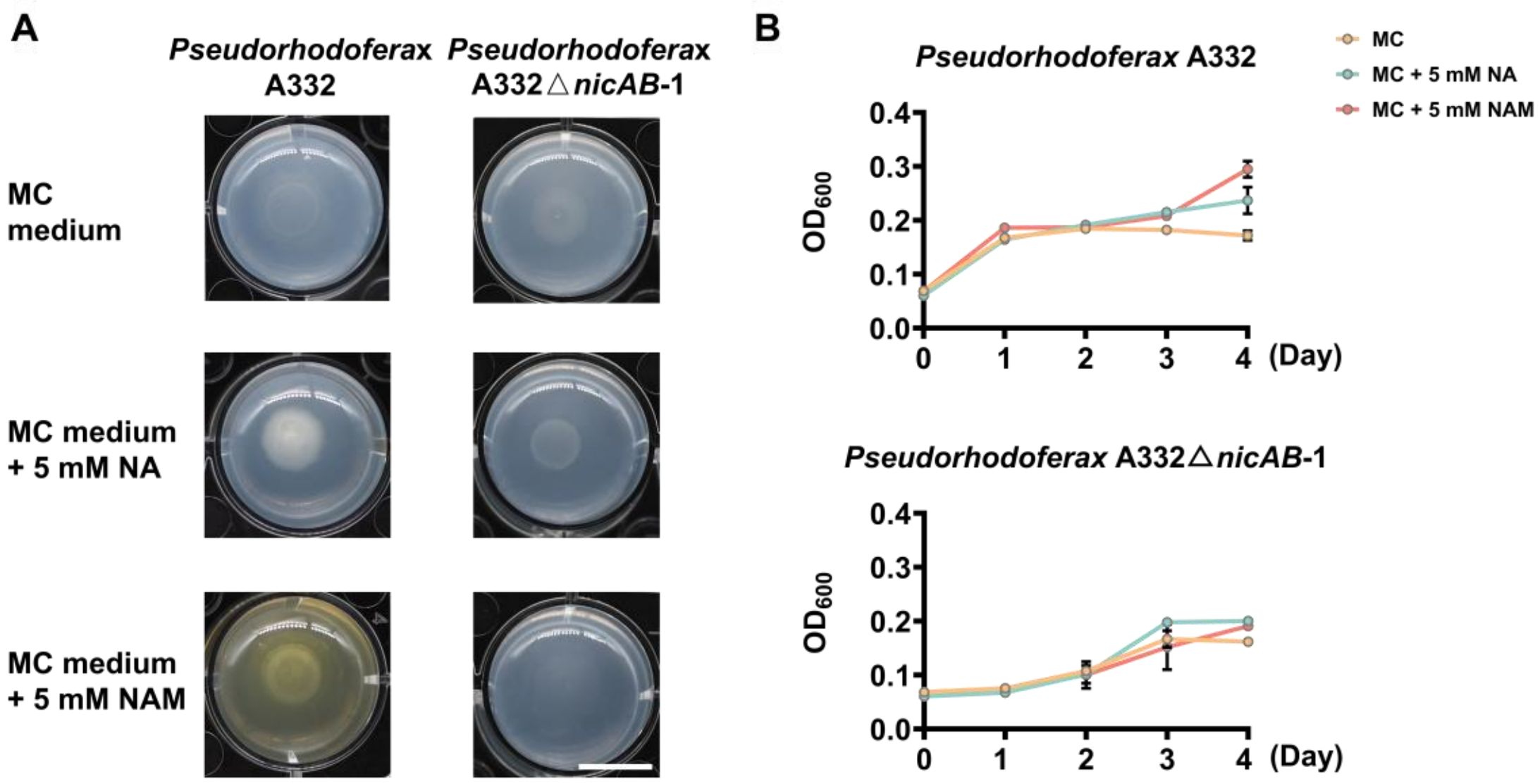

Fig. S17. Deletion of *nicAB* impairs the growth of *Pseudorhodoferax* A332 on NA and NAM supplemented minimal medium.

(A) Growth of wild-type *Pseudorhodoferax* A332 and the Δ*nicAB* mutant on MC minimal medium under basal conditions or supplemented with NA or NAM. Representative images are shown. Scale bar, 1 cm.

(B) Growth curves of the same strains in liquid MC medium under the indicated conditions. Optical density at 600 nm (OD_{600}) was measured daily from day 0 to day 4. Data are presented as mean ± s.d. (n = 4).

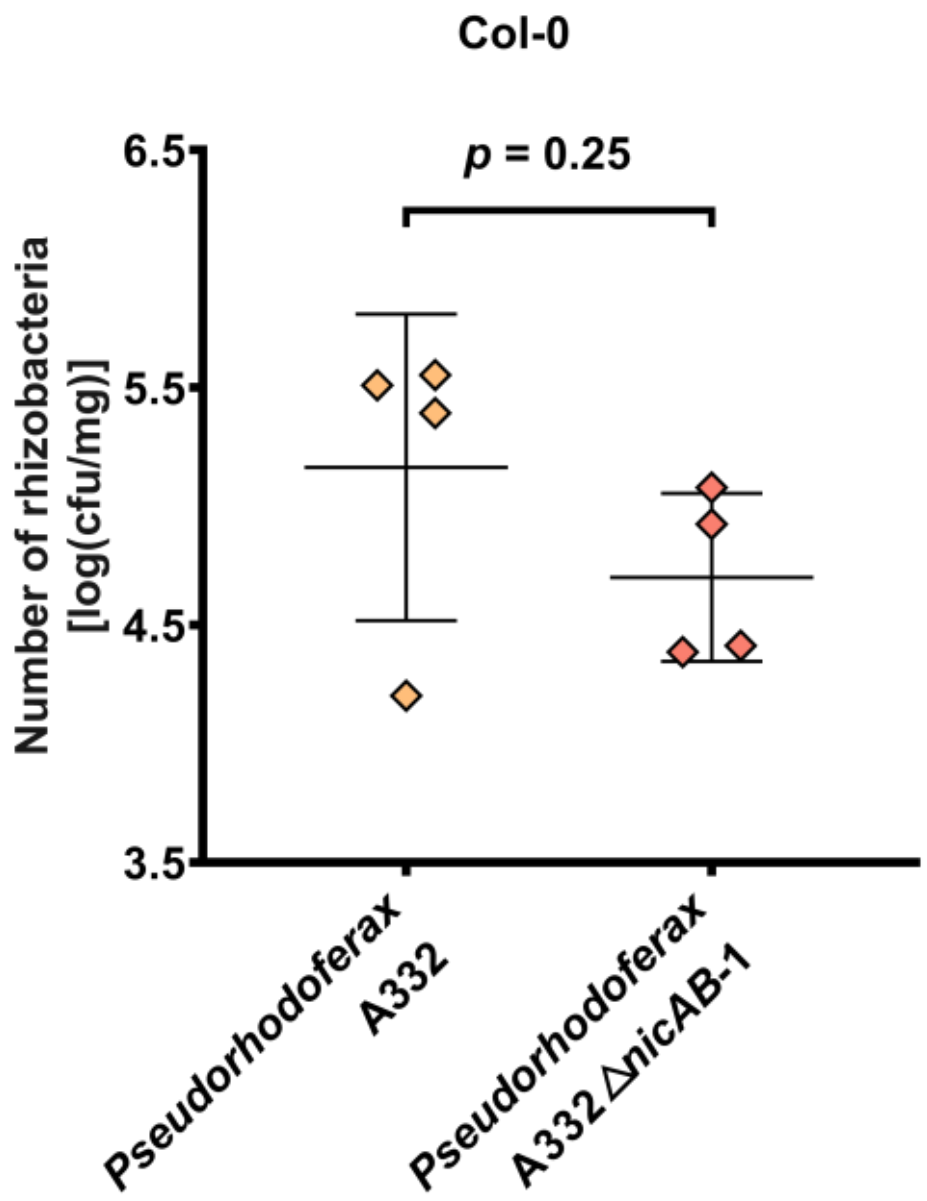

Fig. S18. Root colonization of *Pseudorhodoferax* A332 is comparable between the wild-type and its Δ*nicAB* mutant.

Root colonization levels of wild-type *Pseudorhodoferax* A332 and its Δ*nicAB* mutant in Col-0 plants. All data represent mean ± s.d. (n = 4); p-values were determined by unpaired two-tailed Student's t-tests.

Supplemental Tables

Table S1. Comprehensive metadata and analyses for the study of *Arabidopsis* root microbiota and their correlation with NA-related metabolites, related to Fig. 2.

(A) Root microbiota metadata, ASV abundance table, and representative 16S rRNA gene sequences.

(B) Comparative analysis of root microbiota ASVs in *nic1-1*, *ugt74f2*, and *ugt74f2 ugt76c4* double mutants relative to Col-0.

(C) Spearman correlation statistics between differential ASVs and NA-related metabolites.

(D) Taxonomic classification of 22 bacterial strains identified through correlation network analysis.

Table S2. Integrated spectroscopic and genomic evidence for 6-OHNA structure and bacterial biosynthesis, related to Fig. 3.

(A) ^{1}H and ^{13}C NMR spectroscopic data (δ) for 6-OHNA in DMSO-d_6.

(B) BGCs identified in 441 bacterial strains from the *Arabidopsis* root microbiota collection.

(C) Taxonomic classification of bacterial strains after genomic screening and functional prediction for *nicAB* genes, and putative homologs involved in 6-OHNA biosynthesis.

Table S3. Oligonucleotide primers used in this study.

Table S4. MRM (multiple reaction monitoring) settings for analyzed metabolites.
